# Impact of genistein on the gut microbiome of humanized mice and its role in breast tumor inhibition

**DOI:** 10.1371/journal.pone.0189756

**Published:** 2017-12-21

**Authors:** Bidisha Paul, Kendra J. Royston, Yuanyuan Li, Matthew L. Stoll, Christine F. Skibola, Landon S. Wilson, Stephen Barnes, Casey D. Morrow, Trygve O. Tollefsbol

**Affiliations:** 1 Department of Biology, University of Alabama at Birmingham, Birmingham, Alabama, United States of America; 2 Comprehensive Cancer Center, University of Alabama at Birmingham, Birmingham, Alabama, United States of America; 3 Division of Pediatric Rheumatology, University of Alabama at Birmingham, Birmingham, Alabama, United States of America; 4 Department of Hematology and Medical Oncology, Emory University School of Medicine, Atlanta, Georgia, United States of America; 5 Targeted Metabolomics and Proteomics Laboratory, University of Alabama at Birmingham, Birmingham, Alabama, United States of America; 6 Department of Pharmacology and Toxicology, University of Alabama at Birmingham, Birmingham, Alabama, United States of America; 7 Comprehensive Center for Healthy Aging, University of Alabama at Birmingham, Birmingham, Alabama, United States of America; 8 Nutrition Obesity Research Center, University of Alabama at Birmingham, Birmingham, Alabama, United States of America; 9 Department of Cell, Development & Integrative Biology, University of Alabama at Birmingham, Birmingham, Alabama, United States of America; 10 Comprehensive Diabetes Center, University of Alabama at Birmingham, Birmingham, Alabama, United States of America; University of South Alabama Mitchell Cancer Institute, UNITED STATES

## Abstract

Since dietary polyphenols can have beneficial effects in prevention and treatment of cancer, we tested the hypothesis that breast cancer patients’ intestinal microbiota is modulated by genistein (GE), an isoflavone found in soy, and that microbial alterations may offset the side effects brought about by chemotherapy. We demonstrated successful humanization of germ-free mice by transplanting fecal samples from breast cancer patients before and after chemotherapy. Mice were then grouped based on chemotherapy status and GE or control diet. We did not find any significant differences between pre-chemotherapy and post-chemotherapy bacterial composition and abundances. Germ-free mice on a GE diet showed differences in microbial composition as compared to mice on control diet. Four weeks after introduction of the customized GE diet, there was distinct clustering of GE-fed mice as compared to the control-fed group. In the gut microbiome of GE-treated humanized mice, there was an increase in abundance of genera *Lactococcus* and *Eubacterium*. Phylum Verrucomicrobia showed statistically significant (p = 0.02) differences in abundances between the GE-fed and control-fed groups. There was an increase in bacteria belonging to family Lachnospiraceae and Ruminococcaceae in GE-fed mice. Marked changes were observed in GE catabolism in mice humanized with fecal material from two of three patients’ post-chemotherapy with complete disappearance of 4-ethylphenol and 2-(4-hydroxyphenol) propionic acid conjugates. The post-tumor samples did not show any distinct clustering of the gut microbiota between the two diet groups. There was an increase in latency of about 25% for tumor growth of the humanized mice that were on a GE diet as compared to humanized mice on a control diet. The average tumor size for the GE group was significantly decreased compared to the non-GE group. Collectively, our results suggest that the intestinal microbiota becomes altered with a GE diet before induction of tumor. Our findings indicate that GE modulates the microbiome in humanized mice that may contribute to its effects on increasing the latency of breast tumor and reducing tumor growth.

## Introduction

The three most commonly diagnosed cancers in women are breast, lung and colorectal cancers. Breast cancer is the second leading cause of cancer death among women and accounts for 29% of all new cancer diagnoses in women. According to a recent survey, about 246,660 new cases of breast cancer were diagnosed in the United States in 2016 [1]. About 79% of women diagnosed with estrogen-receptor (ER)-positive [ER (+)] breast cancer receives hormone therapy [2]. ER (-) negative breast cancer patients and those with metastatic tumors or potential are often recommended for surgical removal of the primary tumor followed by aggressive chemotherapy including adriamycin (doxorubicin), cisplatin, cyclophosphamide, paclitaxel or docetaxel [3]. These chemotherapeutic agents cause various side effects that can have both short and long-term effects [4], thus patients are increasingly turning to complementary and alternative medicines (CAM) [5]. One such alternative is the use of natural compounds and several of our studies have indicated the efficacy of certain compounds as adjuvant therapies. For example, in 2013 we found that epigallocatechin gallate (EGCG), a green tea polyphenol, in conjunction with sulforaphane (SFN), a component of cruciferous vegetables, was capable of enhancing the efficacy of cisplatin [6]. In another study, we demonstrated genistein’s (GE) ability to inhibit breast cancer cell growth as well as enhance the efficacy of hormone therapy in ER (-) breast cancer cells [7].

Asian women consume an average of 20–50 times more soy products per capita than their western counterparts and are less susceptible to developing breast cancer [8, 9] which makes this compound an ideal candidate for study. GE is an isoflavone derived from soy products and exerts its anticancer properties through various mechanisms such as apoptosis, inhibition of angiogenesis, DNA methyltransferase (DNMT) inhibition and human telomerase reverse transcriptase (hTERT) suppression [10–12]. Previous studies have shown that GE is effective in breast cancer prevention by binding to estrogen receptors [13] and the bacterial metabolite of the isoflavone daidzein, S (-) equol, shows a greater affinity for estrogen receptor β than daidzein itself [14].

**Fig 1:**
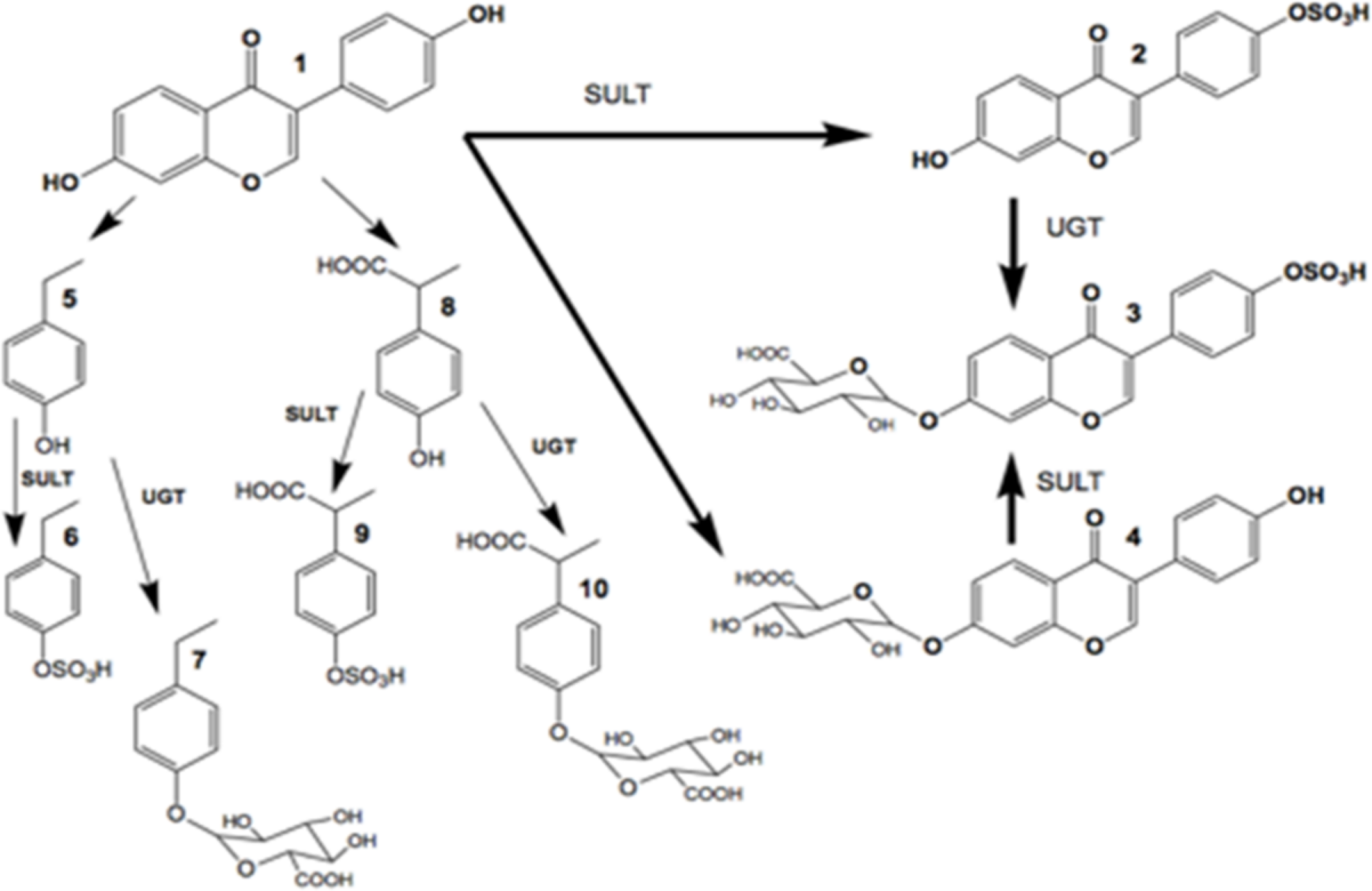
GE metabolism in mice. Schematic drawing of the ways in which GE is metabolized in mice. GE **1** undergoes both sulfate **2** and glucuronide **4** conjugation, as well as cleavage reactions to generate 4-ethylphenol **5** and 2-(4-hydroxyphenyl) propionic acid **8**. Both of these cleaved metabolites undergo sulfate **6, 9** and glucuronide conjugation **7, 10**. SULT = 3’-phosphoadenosine 5’-phosphosulfate sulfotransferase, UGT = uridine 5'-diphospho glucuronosyltransferase.

The human gastrointestinal tract harbors a community of a thousand or more species of bacteria amounting to 10^14^ cells [15] and that is 10 times greater than the number of eukaryotic human cells. However, a recent paper has stated that the ratio may be closer to 1:1 [16]. The intestinal bacteria, mostly anaerobic, produce a number of important metabolites, including short chain fatty acids (SCFA), B-vitamins, folate and biotin, all of which can modulate epigenetic processes that in turn can affect tumorigeneses [17]. Many studies have focused on the role of the microbiome in the etiology of various types of cancer [18]. Some studies have investigated the differences in gut microbiome between breast cancer patients and non-breast cancer patients and have found significant differences in their microbial composition [19]. Previous reports have shown a close correlation of the gut microbiome and colorectal cancer progression, yet few have studied the effects of GE on the microbiome in relation to breast cancer [20, 21]. Some studies have shown that a GE-enriched diet may potentially impact the composition of gut microbiome [22].Our study explores the effect of GE on the gut microbiome of germ-free (GF) mice and its impact on breast cancer prevention and treatment. Bacteria can either interact directly with the host or indirectly via exchange of metabolites with the host [23]. In this study, we performed 16S amplicon sequencing of 16S rRNA genes to analyze the overall structure of microbiota of breast cancer patients before and after chemotherapy. We performed fecal microbiota transplantation (FMT) into the gut of GF mice and evaluated for establishment of a humanized microbiome. We also studied the impact of a GE diet on the microbiota on the humanized mice as well as whether the human microbiota altered GE metabolism (Fig 1). In addition, we induced ER (-) breast orthotopic xenografts in these mice and assessed the effect of GE on tumor growth and volume in GF mice transplanted with human fecal microbiota. Moreover, we identified key bacterial phylotypes that change significantly with dietary changes and tumor induction.

## Materials and methods

### Ethics statement for obtaining patient samples

The study enrolled breast cancer patients in the age range of 37–66 years in accordance with institutional IRB requirements. We gathered the following information from the patients: age, race, diagnoses, size, grade, metastases, ER/progesterone receptor (PR)/ human epidermal growth factor receptor 2 (HER) statuses, and chemotherapy regimen. Fecal samples were collected prior to and after doxorubicin and docetaxel-based chemotherapy. Samples were de-identified for the purpose of experimentation. Since the patients voluntarily shipped the fecal samples after chemotherapy, there was a variation in time (2–4 weeks) post-chemotherapy. For our pilot study, we analyzed patient samples (n = 3) before and after chemotherapy [Table 1]. All individuals provided written informed consent prior to participating in the study. All study protocols were approved by the Ethics Committee of the University of Alabama at Birmingham.

**Table 1:** Patient summary.

Patients	Patient 1	Patient 2	Patient 3
Age	66	52	37
Race	White	White	White
Diagnosis	Invasive Ductal Carcinoma	Invasive Ductal Carcinoma	Invasive Ductal Carcinoma
Node	Yes	No	No
Metastasis	Yes	No	No
ER/PR	Positive	Negative	Positive
HER	Negative	Negative	Positive
BRCA	Not indicated	Not indicated	Positive
Grade	3	3	1
Chemotherapy	Docetaxel	Doxorubicin	Docetaxel

### Sample collection and DNA extraction

The patients were asked to provide samples voluntarily. The samples were collected in modified Cary Blair [24] medium and sent via FedEx Clinical Pak. The fecal samples were diluted in Cary Blair medium to 0.1 mg/mL for a total volume of 20 mL with 10% by volume glycerol. Aliquots of 5 ml were dispensed into cryovial tubes and stored at -80°C. Genomic DNA was extracted from tissue and swab samples by bead-beating using the Fecal DNA Isolation Kit from Zymo Research (Irvine, CA, USA) according to manufacturer’s instructions. The isolated DNA was immediately used for PCR or stored in standard Tris-EDTA buffer (pH 8) at 4°C. Prior to PCR, the isolated PCR DNA was quantified using a microspectrophotometer (ThermoFisher, Waltham, MA) [25].

### 16S V4 rRNA gene sequencing and bioinformatic analysis

Once the sample DNA was prepared, an amplicon library was created from individual samples using PCR with uniquely barcoded primers to amplify the V4 region of the 16S rRNA gene [25]. Illumina MiSeq [26] was used to sequence the PCR products with paired-end reads of about 250 bp from the V4 region of the 16S rRNA gene. The oligonucleotide primers used for the PCR amplification of the V4 region of the 16S rRNA gene were as follows (Eurofind Genomics, Inc., Huntsville, AL): Forward V4:

5’-AATGATACGGCGACCACCGAGATCTACACTATGGTAATTGTGTGCCAGCMGCCGCGGTAA-3’; and

Reverse V4:

5’-CAAGAGAAGACGGCATACGAGATNNNNNNAGTCAGTCAGCCGGACTACHVGGGTWTCTAAT-3’.

Sample quantification was performed using PICO green dsDNA Reagent. Raw Illumina fastq files were de-multiplexed, quality filtered and analyzed using Quantitative Insight into Microbial Ecology (QIIME) [27] data analysis package. Uclust clustering program was used to group the sequences into Operational taxonomic unit (OTU) with 99% similarity. The RDP has been trained using Greengenes (version 13.8) [28] and was used to make taxonomic assignments. Multiple sequence alignment of OTUs was performed with PyNAST [29]. Alpha diversity was calculated with Chao1 [30, 31] and Phylogenetic Diversity [32], and beta diversity was calculated with weighted Unifrac [33] and Bray Curtis [34].

### Animals

All animal studies were approved by the Institutional Animal Use and Care Committee of the University of Alabama at Birmingham (UAB). Germ-free RAG2^-/-^ (Taconic Biosciences Inc., Hudson, NY) athymic female mice (6-weeks age) were used for our studies for the purpose of performing xenografts. Mice were bred in the Animal Facility at the University of Alabama at Birmingham (UAB). Mice were maintained in semi-rigid isolators that were housed within the UAB Gnotobiotic Facility, within a 12-hour light/dark cycle and an ambient temperature of 23 ± 1°C. The isolators were regularly sterilized with Exspor (Alcide, Redmond, WA). Animals had free access to food (control irradiated diet without GE) and water.

### Diet and colonization of mice

Mice were housed by human sample chemotherapy status and diet [Table 1] (4 mice/ cage- a total 48 mice in 12 cages). Fecal suspension (200 μl) was transplanted into mice using oral-gavage and enema routes [35]. Co-housing of four GF mice with one colonized mouse led to successful colonization of all four GF mice. After four weeks, mice were divided into two major groups and were fed either GE diet or control diet in the form of pellets. Mice on the GE diet were fed a special corn oil customized diet from Teklad/Envigo (TD. 140534-GE 0.25 g/Kg) and the mice on the control diet were fed a special corn oil diet without GE (TD 140535). Teklad vitamin mix was used to make the diet more suitable for irradiation. Teklad/Envigo supplied all diet except GE powder which was supplied by LKT laboratories, St. Paul, MN (purity ≥98%). Mice food was in the form of pellets. Genistein supplemented diet as well as control diets were sterilized by irradiation (20-50kGY) as recommended by Teklad Envigo [36].

### Tumor induction

**Fig 2:**
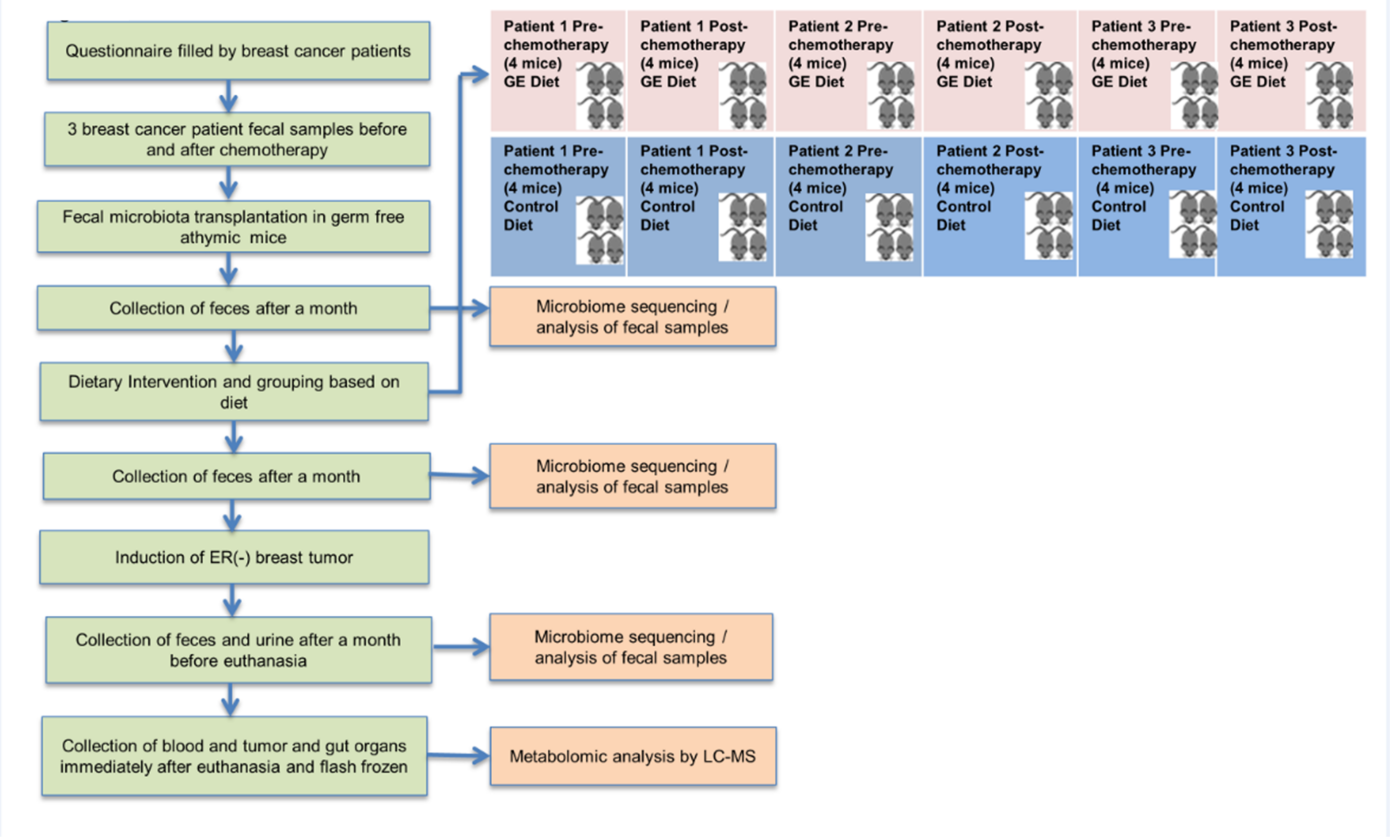
Study design flowchart. Breast cancer patients on chemotherapy (DOX and docetaxel) were recruited for the study. Fecal samples were obtained from three breast cancer patients before and after chemotherapy and transplanted into germ-free athymic mice. Feces were collected from mice after a month and compared with fecal donor samples by performing microbiome analysis. The second stage of the project involved grouping mice based on diets. Mice were fed GE diet or control diet and grouped into different cages based on diet and chemotherapy as shown. Feces were collected from mice two weeks and four weeks after start of diet and fecal microbiome analysis was performed to compare the microbiome changes in the distinct groups. The third stage of the project involved induction of ER (-) breast tumor in mice. Feces and urine were collected after tumor size reached 1 cm^3^ one day before euthanization. On the day of euthanization, blood and tumor were collected and flash frozen for metabolomic and epigenetic analysis.

Breast cancer orthotopic xenografts were established in both groups of mice. An aliquot (100 μl) of exponentially growing ER (-) breast cancer MDA-MB-231 cells (2 x 10^6^) was injected orthotopically into the second or third pair of the right thoracic mammary fat pad of each GF mouse to determine the *in vivo* efficacy of these diets on human breast tumor development. Only one inoculation per mouse was performed. Four-weeks after injection of tumor in both control and GE-fed diet mice groups, the tumor size reached a volume of 1.0 cm^3^. Fecal and urine samples were collected one day before euthanization of mice. Serum and tumor samples were collected immediately after euthanization (Fig 2).

### Sample preparation

Pooled urines (50 μL) from the 12 mice groups (Table 1) were mixed with 200 μl ice-cold methanol (4:1, by volume) with shaking for 30 min. The precipitated proteins were removed by centrifugation at 14,000 x g for 10 min at 4°C. The supernatants were carefully decanted and taken to dryness under N_2_. The dried extracts were re-suspended in 500 μL double-distilled H_2_O with 0.1% formic acid and centrifuged at 14,000 x g for 10 min at 4°C.

### LC-MS/MS analysis

A clear aqueous solution of the urine extract (5 μl) was injected onto an Eksigent reverse-phase C_18_ pre-column cartridge (0.5 cm x 200 μm ID) in 0.1% formic acid. Metabolites bound to the cartridge were eluted with a 20-min linear gradient of 0–80% acetonitrile in 0.1% formic acid onto an Eksigent ChipLC C_18_ column (15 cm x 200 μm ID) at a flow rate of 1 μl/min. The ChipLC column was placed in an Eksigent Nanoflex (SCIEX, Concord, ON, Canada) operating at 45°C. At the end of each analytical run, the column was washed with acetonitrile:0.1% formic acid for 1 min followed by re-equilibration with 0.1% formic acid for 4 min. Eluate from the ChipLC column was passed into the nanoelectrospray ionization interface of a SCIEX Triple TOF™ 5600 Mass Spectrometer System operating in the negative and positive ion modes. The collision energy was set to 35 eV with a 15 eV collision spread, curtain gas to 20, GS1 to 15, spray voltage to 2300 V (positive ion mode)/2200 V (negative ion mode), and temperature to 120°C. In each duty cycle lasting 1.25 s, high mass resolution MS spectra were collected for 250 ms followed by 50 ms MS/MS spectra of the top 20 most intense molecular ions. Ions producing successful MS/MS spectra were put onto an exclusion list for the next 60 s.

### Data processing

The collected data (in.wiff format) were converted to.mzxml format using MSConvert (provided by Proteowizard - http://proteowizard.sourceforge.net/downloads.shtml)). The.mzxml files were analyzed using MZmine 2.28 (https://github.com/mzmine/mzmine2/releases)). The following negative ion chromatograms were created for GE and its known metabolites [37, 38]: GE (C_15_H_10_O_5_) *m/z* 269.0435; GE sulfate (C_15_H_10_O_8_S) *m/z* 349.0016; GE- β-glucuronide (C_21_H_18_O_11_) *m/z* 445.0765; GE-β–glucuronide/sulfate (C_21_H_1_ O_14_S) *m/z* 525.0333; 4-ethylphenyl sulfate (C_8_H_10_O_4_S) *m/z* 201.0216; 4-ethylphenyl-β-glucuronide (C_14_H_18_O_7_) *m/z* 297.0968; 2-(4-hydroxyphenyl) propionic acid (C_9_H_10_O_3_) *m/z* 165.0546; 2-(4-sulfoxyphenyl) propionic acid (C_9_H_10_O_6_S) *m/z* 245.0114; 2-(4-β-glucuronylphenyl) propionic acid (C_15_H_18_O_9_) *m/z* 297.0867. Relative quantification of each metabolite was based on peak heights since in the case of GE sulfate, multiple occluded peaks were observed. The peaks assigned to each metabolite were assigned based on their MSMS spectra and their absence in urines from mice not receiving GE in their diet.

### Statistical analysis

Student’s t test was performed using Microsoft Excel. P<0.05 was used as the cut-off value for statistical significance. Heatmaps of microbiome data (OTUs) were made using the heatmap command in R. The heatmap command reorders the data in the rows, and the columns separately, so that similar data are grouped together by hierarchical clustering. Heatmaps of the bacterial taxa were constructed based on the relative abundance of each bacterial strain in each sample for both diet groups.

## Results

### Microbiota profile of human and murine fecal samples after humanization

**Fig 3:**
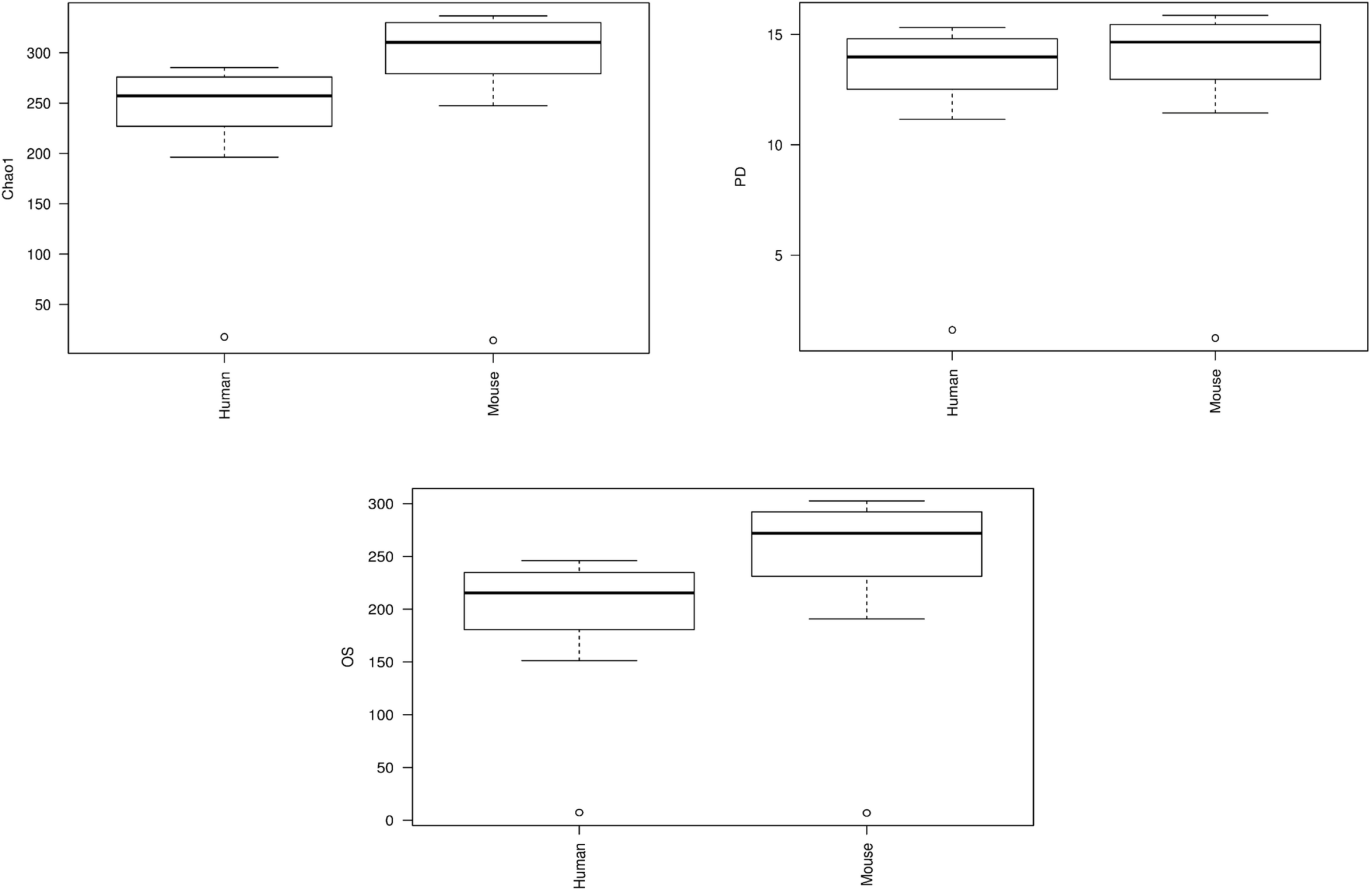
Humanization of mice. Alpha diversity represented in the form of box plots show similar richness and evenness in humanized mice and human donors four weeks after humanization of the mice using FMT. The phylogenetic diversity (PD) evaluates the length of all branches of the phylogenetic tree of a given population. Chao1 estimates the total species richness and observed species (OS) counts the unique OTU’s. The median, the first quartile and third quartile values in humans resemble that of mice. Whiskers in the boxplot represent the range of minimum and maximum alpha diversity values within a population, excluding outliers. The range of values for human and mice are similar. These results indicate that the summary statistics (measures of central tendencies) of human and mice data are alike further enforcing the fact that the humanization of mice was successful.

As indicated in the study design, we analyzed the microbiome of breast cancer patients (Fig 2). Fig 3 shows alpha diversity matrices; chao1, observed species and PD whole tree. There were no statistically significant differences between the mice and the human donors in all of these matrices. Overall species richness of mouse and human microbiota evaluated with the Chao1, phylogenetic diversity and observed species metrics, was compared with the Student's t-test and visualized with boxplots (Fig 3). Richness in mice was very similar and almost overlapped that of human donors. The shape of the curve flattened (0 instantaneous slope) at the last point of sampling indicating the observance of all taxonomic units. The fecal microbial transplantation (FMT) resulted in the GF RAG2^-/-^ mice having acquired a similar composition as compared to the human donors. The GF mice and human donors had similar percentages of Actinobacteria (humanized mice 13% vs mice 12%). The abundance of Firmicutes in mice was less than that of humans, although there was no significant difference between their mean abundances (p = 0.21). The phylum Verrucomicrobia showed similar abundance in both human as well as mice (p = 0.96). Overall, our results showed that humanization was successful with no significant differences in alpha diversity of the mouse and human samples.

### Taxonomic differences between the control and GE-fed diet groups prior to the induction of tumor

**Fig 4:**
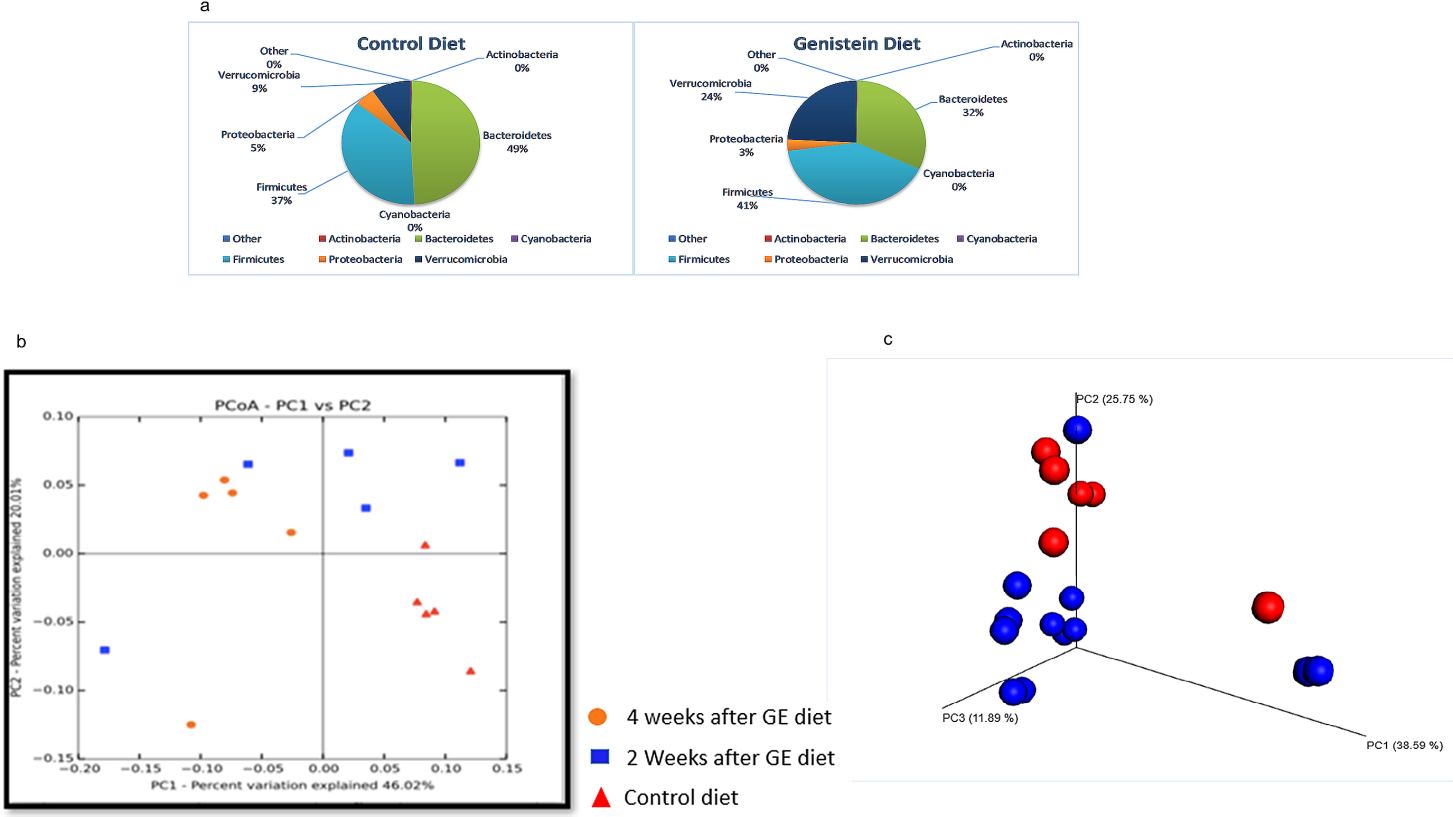
Microbiota change four weeks after introduction of GE diet. (a) Phylum level changes in microbial abundance four weeks after introduction of GE diet. There was a significant increase in Verrucomicrobia (p = 0.02) in mice on the GE diet. (b)Weighted unifrac 2. (c) 3D PCoA plots (Bray Curtis) showing a distinct clustering of the control-fed (red) and GE-fed diet groups (blue).

**Fig 5:**
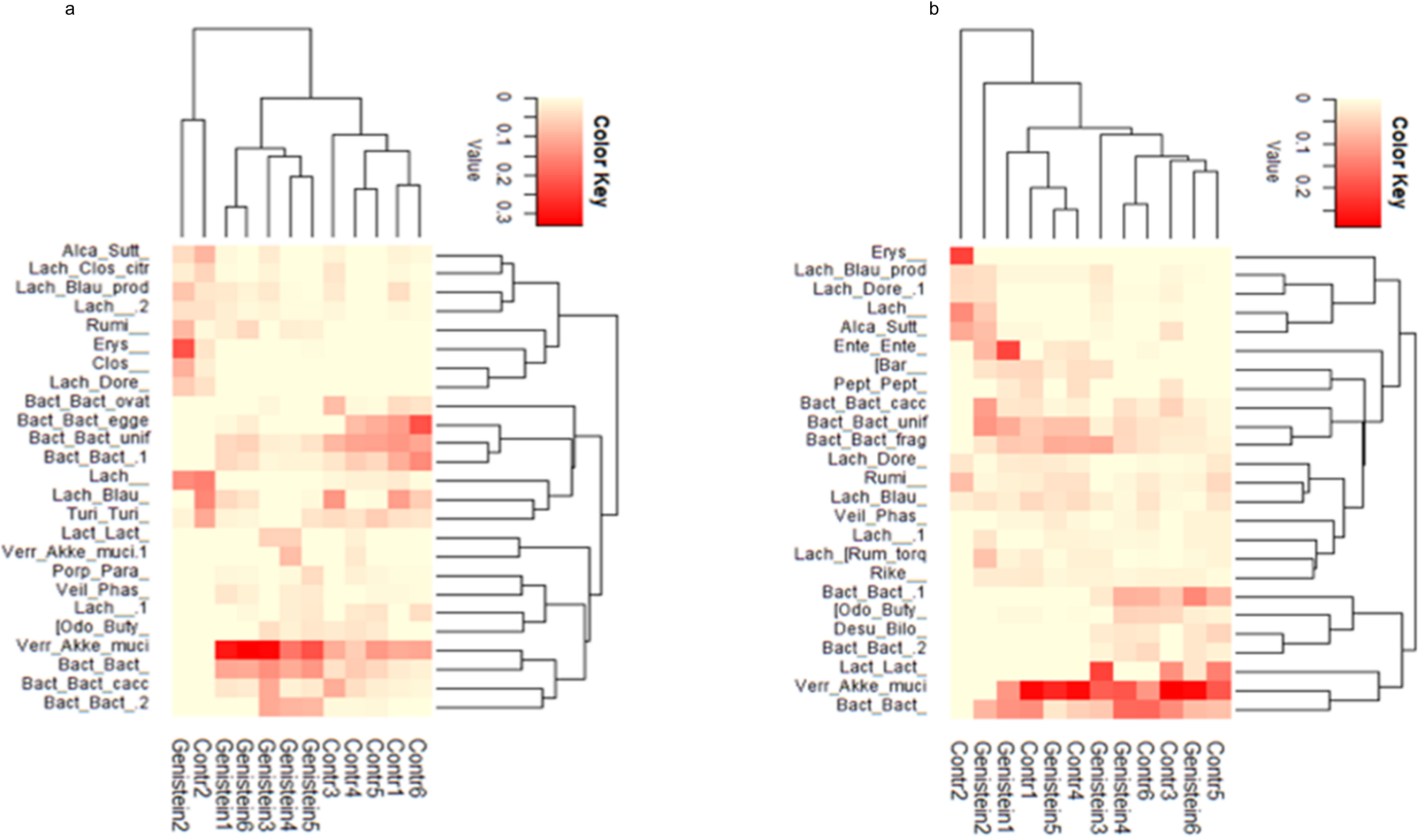
Heat maps showing relative abundance of bacteria before and after tumor induction. (a) Heat map of the pre-tumor microbial abundance between control and GE groups. (b) Heat map of the post-tumor microbial abundance between control and GE diet groups. Dendogram shows a distinct clustering of GE-fed group vs control-fed group. Red color denotes high abundance; yellow color denotes low abundance. Post-tumor abundances are shown in Table 3. The lineages are abbreviated, the details of which are given in S1 and S2 Tables.

At the phylum level, the two diet groups showed differences in microbial abundances (Fig 4A). The control group of humanized mice showed greater abundances of Bacteroidetes as compared to the humanized mice on the GE diet (49% versus 32%, p = 0.18). There was a significant increase in Verrucomicrobia in the GE-treated group of mice (9% versus 24%, p = 0.021). The beta diversity weighted Unifrac 2-D plot showed distinct clustering of the two diet groups (Fig 4B). The groups showed differences in clustering between the controls and two-weeks post start of the GE diet, further clustering evident in the same mice four-weeks post induction of diet. The 3-D PCoA plot distance metric (Bray Curtis) also revealed a distinct clustering of the two diet groups at four weeks (Fig 4C). To validate our observation, we performed Permutation Multivariate Analysis of Variance (PERMANOVA) from the distance matrix of the Bray Curtis test of beta diversity (F = 3.17, p = 0.011). Fig 5 shows the heat map with a dendogram depicting the relative abundance of the top 25 taxonomic units. Compared to the control diet, the relative abundance of Verrucomicrobia (f_Verrucomicrobiaceae g_*Akkermansia* s_*muciniphila*) was higher in the GE-fed group of humanized mice [Fig 5A, S1 Table]. Furthermore, it was observed that Bacteroidetes (g_*Bacteroides* s_unclassified) and family Erysipelotrichaceae showed higher relative abundance in the mice receiving the GE-fed diet. Table 2 shows the bacterial taxa that were significantly different between the mice for the GE-fed and control-fed groups after introduction of the GE diet. Bacteria of order Lactobacillales and family Enterococcaceae increased 20-fold in mice on the GE-fed diet. Some species of bacteria that increased significantly after consumption of the GE diet were *Eubacteriumdolichum*, *Akkermansia municiphila*, *Ruminococcus torques and Clostridium hathewayi*. The species of bacteria that were significantly lower in the GE-fed group compared to control-fed group were *Bacteroides eggerthii* and *Bacteroides ovatus*. Overall, our results showed a significant decrease in members of genus *Paraprevotella*, *Anaerostipes*, *Bacteroides* (species *uniformis*, *eggerthii* and *ovatus*), *Turicibacter*, *Blautia* and *Coprobacillus*, while there was a significant increase in genus *Lactococcus*, *Akkermansia* and *Eubacterium*.

**Table 2:** Bacterial species showing significant changes in relative abundance after introduction of GE diet.

Phylum	Class	Order	Family	Genus	Species	Abundance (Control Diet)	Abundance (Genistein Diet)	p-value (t-test)
Firmicutes	Bacilli	Lactobacillales	Streptococcaceae	Lactococcus		0.00%	0.64%	5.32784E-06
Firmicutes	Erysipelotrichi	Erysipelotrichales	Erysipelotrichaceae	[Eubacterium]	dolichum	0.07%	0.77%	0.002855366
Firmicutes	Bacilli	Turicibacterales	Turicibacteraceae	Turicibacter		5.39%	1.29%	0.011877701
Bacteroidetes	Bacteroidia	Bacteroidales	Bacteroidaceae	Bacteroides	uniformis	9.73%	2.98%	0.01193515
Bacteroidetes	Bacteroidia	Bacteroidales	Bacteroidaceae	Bacteroides		3.36%	10.24%	0.01507205
Verrucomicrobia	Verrumicrobiae	Verrumicrobiales	Verrumicrobiaceae	Akkermansia	muciniphila	8.69%	22.62%	0.029623048
Firmicutes	Clostridia	Clostridiales	Lachnospiraceae			0.18%	0.80%	0.034124965
Bacteroidetes	Bacteroidia	Bacteroidales	Bacteroidaceae	Bactoides	eggerthii	9.01%	0.48%	0.034429239
Firmicutes	Bacilli	Lactobacillales	Enterococcaceae			0.05%	0.82%	0.03585814
Firmicutes	Clostridia	Clostridiales	Ruminococcaceae			0.20%	3.35%	0.039400762
Proteobacteria	Gammaproteobacteria	Enterobacteriales	Enterobacteriaceae			0.02%	0.11%	0.040164148
Firmicutes	Clostridia	Clostridiales	Lachnospiraceae	Blautia		8.01%	1.51%	0.045133043
Firmicutes	Clostridia	Clostridiales	Veillonellaceae	Phascolarctobacterium		0.38%	1.63%	0.054393931
Firmicutes	Clostridia	Clostridiales	Ruminococcaceae	Ruminococcus		0.08%	0.12%	0.060319728
Bacteroidetes	Bacteroidia	Bacteroidales	Bacteroidaceae	Bactoides		0.47%	4.94%	0.073152923
Bacteroidetes	Bacteroidia	Bacteroidales	Bacteroidaceae	Bactoides	ovatus	2.93%	0.26%	0.073646339
Bacteroidetes	Bacteroidia	Bacteroidales	Bacteroidaceae	Bactoides		6.68%	2.15%	0.080005155
Proteobacteria	Gammaproteobacteria	Enterobacteriales	Enterobacteriaceae	Escherichia	coli	0.08%	0.53%	0.100722323

### Taxonomic differences between GE-fed and control-fed groups post induction of tumor

**Fig 6:**
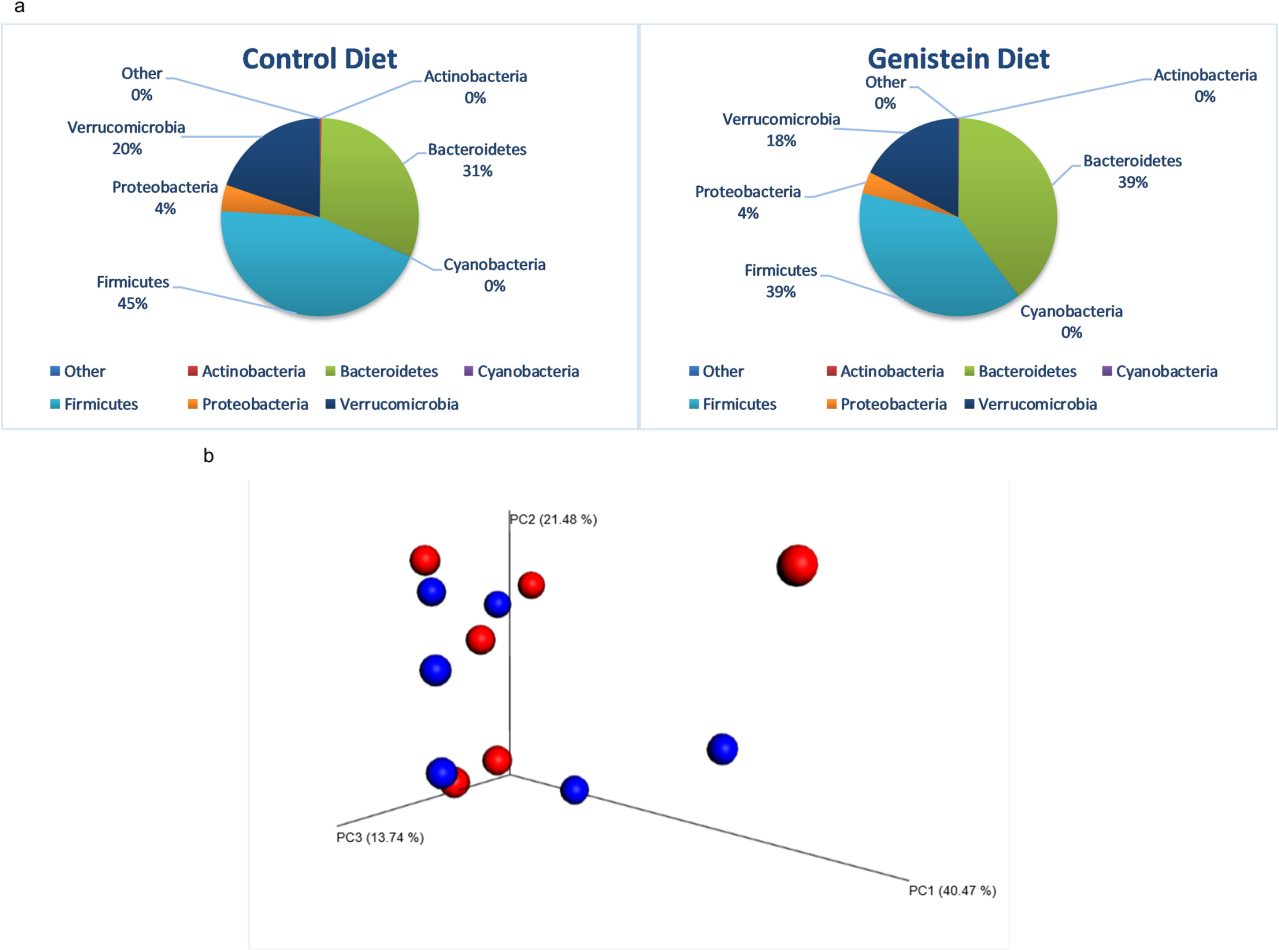
Microbiota change four weeks after induction of tumor. (a) Phylum level changes in microbial abundance after induction of tumor. There was no distinct difference in microbial composition between control and GE diet groups. (b) 3D-PCoA plot (Bray Curtis) does not show any distinct clustering of control-fed (red) and GE-fed mice (blue).

After tumor induction, the microbial composition of the GE-fed group of humanized mice resembled that of the control group. The distinct grouping of the two diet groups was lost after induction of tumor. Fig 6A shows the phylum level abundances of the two diet groups. The percentages of different phyla of bacteria are similar in the two groups. The percentage of Bacteroidetes in the GE-fed group was 39% and control group was 31% (p = 0.33) and for Verrucomicrobia, it was 39% and 45%, respectively (p = 0.74). The heat map (Fig 5B) shows the abundance of f_Verrucomicrobiaceae g_*Akkermansia* s_*muciniphila* to be high in both diet groups and with no distinct dissimilarity. Family Erysipelotrichaceae showed a similar relative abundance in mice in the GE-fed diet group. The 3-D PCoA Bray Curtis plot further illustrates the loss of microbial clustering previously established by the GE-fed diet (Fig 6B). PERMANOVA test on the 3-D PCoA Bray Curtis test supported our observation (F = 0.474, p = 0.918). Though there was no separate grouping of the diet groups, Table 3 displays the bacterial species that were significantly different between the GE-feeding and control-feeding after induction of tumors. There was a decrease in bacteria of genus *Adlercreutzia* (p = 0.01), *Peptococcus* (0.14), *Eubacterium* (species *dolichum* p = 0.16) and *Blautia* (p = 0.18) in the GE-fed diet group when compared to control group. There were increases in bacteria of family Desulfovibrionaceae (p = 0.2), genus *Bacteroides* (species *uniformis*, p = 0.16) and genus Odoribacter. Our results show that about a month after induction of tumor, the bacterial clustering that was previously established due to the GE diet was lost.

**Table 3:** Bacterial species showing significant changes in relative abundance after induction of tumor.

Phylum	Class	Order	Family	Genus	Species	Abundance (Control Diet)	Abundance (Genistein Diet)	p-value (t-test)
Actinobacteria	Coriobacteriia	Coriobacteriales	Coriobacteriaceae	Adlercreutzia		0.19%	0.07%	0.011357448
Firmicutes	Clostridia	Clostridiales	Ruminococcaceae			3.31%	1.36%	0.088634464
Firmicutes	Clostridia	Clostridiales	Lachnospiraceae	Blautia		0.43%	1.08%	0.090382802
Bacteroidetes	Bacteroidia	Bacteroidales	Bacteroisaceae	Bacteroides		0.08%	0.19%	0.09038958

### GE metabolism

**Fig 7:**
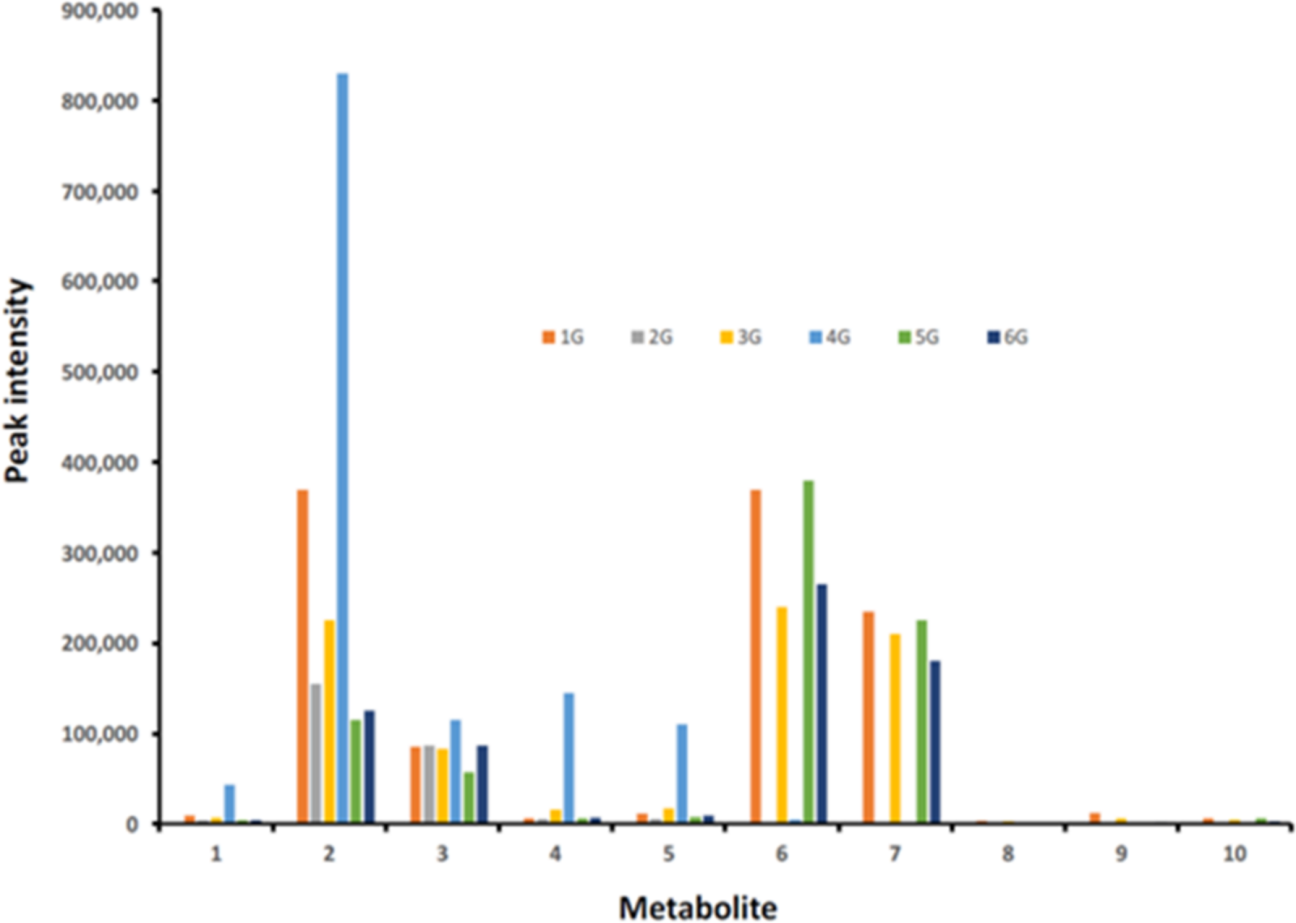
Peak intensities of GE and its metabolites in urines of mice. The nominal masses of the GE metabolites are: *m/z* 269 –GE (**1**), *m/z* 349 –GE sulfate (**2**), *m/z* 445—GE β-glucuronide (**3**), *m/z* 525—GE sulfate/β-glucuronide (**4 and 5**), *m/*z 201–4-ethylphenyl sulfate (**6**), *m/z* 297–4-ethylphenyl β-glucuronide (**7**), *m/z* 165–2-(4-hydroxyphenyl) propionic acid (**8**), *m/z* 245–2-(4-sulfoxyphenyl) propionic acid (**9**), *m/z* 341 2-(4-β-glucuronylphenyl) propionic acid (**10**). G denotes the cages with mice on GE-containing diet. Mice were treated with patient fecal material obtained before (#s 1G, 3G, 5G) and after (#s 2G, 4G and 6G) chemotherapy.

In each of the mice treated with GE, the major urinary metabolites were GE phase II conjugates, GE sulfate (*m/z* 349.0017) and GE β-glucuronide (*m/z* 445.0765), and GE sulfate/β-glucuronide double conjugate (*m/z* 525.0344) (Fig 7). Each of these conjugates had MSMS spectra consistent with their chemical forms. There was no evidence of disulfate or di-β-glucuronide double conjugates in these mouse urines as noted by Soukup et al.[38]. The sulfate/β-glucuronide double conjugate of GE was particularly elevated in mouse #4 humanized with fecal material from a patient after treatment with DOX. The extracted ion chromatogram (EIC) of *m/z* 349.0017 contained at least two unresolved components (particularly for mouse #4), presumably positional isomers of GE-sulfate. The EIC of *m/z* 445.0765 had two chromatographically separated peaks, both with MSMS spectra that were consistent with their being GE-β-glucuronides (principally loss of the β-glucuronide group). In addition to GE conjugates, there was evidence of similar conjugates of dihydrogenistein (*m/z* 271.0601), but at an order of magnitude lower intensities than GE conjugates. No evidence was obtained for sulfated tetrahydroxyisoflavones (*m/z* 364.9961) or their glutathione conjugate (*m/z* 574.1126).

Besides GE conjugates, sulfate and β-glucuronide conjugates of the GE catabolites 4-ethylphenol and 2-(4-hydroxyphenyl)-propionic acid were present in each of the mice humanized with fecal material from patients prior to chemotherapy (Fig 7). The peak intensities of the conjugates of 4-ethylphenol were considerably larger than those of 2-(4-hydroxyphenyl)-propionic acid. The peak intensities of conjugates of 4-ethylphenol were completely absent in two of the three mice humanized with fecal material from patients with grade 3 breast cancer after chemotherapy with docetaxel and doxorubicin (Fig 7). In the mice humanized by fecal material from the third patient with grade 1 breast cancer and treated with docetaxel, conjugates of 4-ethylphenol and 2-(4-hydroxyphenyl)-propionic acid were similar to those humanized with fecal material prior to chemotherapy (Fig 7).

### Tumors

**Fig 8:**
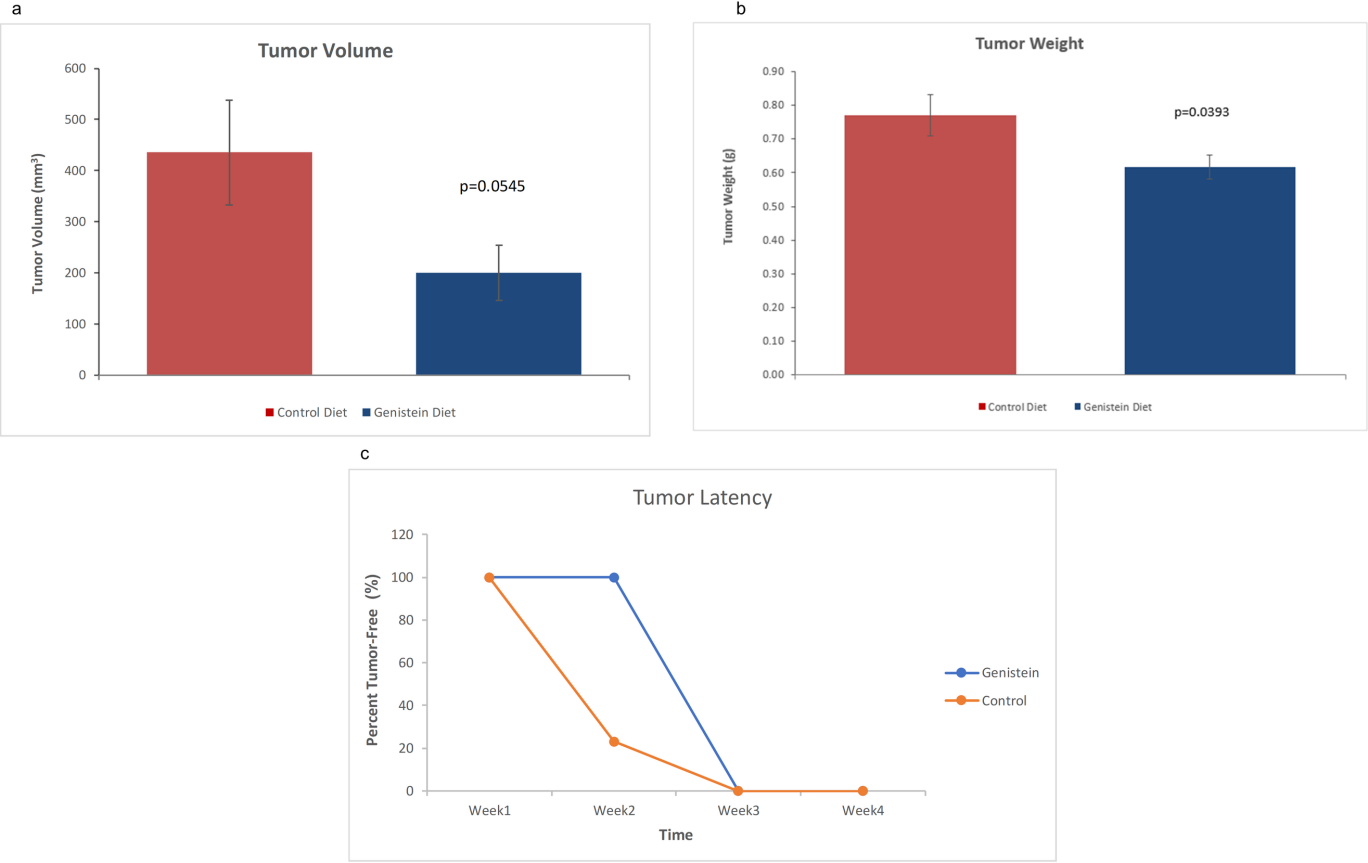
Tumor volume, weight and latency comparisons between control and GE-fed diet groups. Female athymic nude mice were injected with MDA-MB-231 cells. GE or control diets were provided from four weeks prior to injection. (a) GE diet mice showed a nearly significant reduction in tumor volume. (b) GE diet mice showed a significant reduction in tumor weight relative to the control-fed group (n = 18/group). (c) Dietary GE increased the latency of tumor development by 25% for the humanized mice that were on a GE diet as compared to humanized mice on a control diet.

Fig 8 shows the differences in tumor weight, volume and latency between the two diet groups. The humanized mice group that had been fed the GE diet had a nearly significant reduction in tumor volume (p = 0.0545) (Fig 8A). There was a significant reduction in tumor weight (p = 0.0393) for the GE-fed diet group as shown in Fig 8B. The mice on a GE diet incurred a latency of about 1 week in tumor growth (Fig 8C). Overall, the GE-fed group showed a greater percentage of tumor-free mice in the first week. The tumor weight and volume was also significantly less compared to the control group suggesting that GE exhibited its antitumor properties and was successful in decreasing tumor growth.

### FMT pre-chemotherapy vs FMT post-chemotherapy patient fecal sample analysis

The composition of the microbiota in FMT pre-chemotherapy patients when compared to FMT post-chemotherapy patients was similar. Comparison between the two chemotherapy groups for pre-tumor experiments yielded significant results for control-based and GE-based diet subgroups at a 10% level of significance (p = 0.08) for *Bacteroides caccae*. Four weeks after tumor induction, the post-chemotherapy mice group showed significantly lower (10% level of significance) percentage of *Lactobacillus* as compared to pre-chemotherapy samples (p = 0.07) (S3 and S4 Tables).

## Discussion

The role of microbes in breast carcinogenesis has been of great interest. We investigated a potential association of bacterial changes brought about by dietary intervention with tumor prevention and latency. Recent studies have revealed that the interactions between bacteria and host can be very complex [39]. Firstly, the composition of bacterial communities as well as their relative abundances can inform about health status and disease conditions [40]. Secondly, the presence of certain bacteria may have beneficial effects on the body that help to maintain health status [41]. As in the gut, the presence of specific bacteria may be beneficial in the breast.

Our study showed successful humanization of the mice with gut microbiota from patients with breast cancer patients. Surprisingly, there was no significant difference between the microbiota composition of pre-chemotherapy and post-chemotherapy patient samples. This may be due to the small number of patients’ samples analyzed in this pilot study, the time the fecal specimens were collected after chemotherapy and the dosage of chemotherapy. In the future, it would be interesting to expand patient numbers and to collect fecal samples with a larger margin of time before and after chemotherapy. We may find significant changes in biodiversity after a larger time span following chemotherapy. After introduction of the GE-fed diet, there was a microbial alteration that could be observed two weeks and four weeks after introduction of the diet where the relative abundance of some of the dominant phyla changed. After introduction of the GE diet in the mice, certain bacteria such as *Lactococcus*, *Eubacterium dolichum*, *Turicibacter*, *Bacteroides uniformis*, *Akkermancia muniphila* showed significant differences between GE-fed and control diet groups. Very few studies have focused on the association between gut microbiota and breast cancer. A recent review highlights the importance of alteration in gut microbiome in estrogen-modulated diseases [42]. This review suggests that the impact of gut microbiome extends beyond the gut through metabolic changes induced by the gut microbiome. In our study, after tumor induction, the bacteria lost a significant amount of the clustering based on diet groups, though there was a decrease in tumor weight and volume. Loss of microbial clustering may be linked to an increase in tumor size. However, further studies need to be conducted before drawing any conclusions about the direct link between loss of microbial dysbiosis with tumorigenesis.

Table 2 shows that after consumption of GE for about a month in the absence of injected breast cancer cells there was a significant increase in genus *Lactococcus*. Previous studies have shown that *Lactococcus lactis* inhibits the proliferation of SNU-1 human stomach cancer cells through induction of G0/G1 cell cycle arrest and apoptosis via p53 and p21 expression [43]. Another study has shown the health-promoting abilities of *Lactococcus* that can convert mannitol to fatty acids and thus protect humans from colorectal cancer [44]. There was also a significant increase in family Lachnospiraceae both pre- and post-tumor and an increase in family Ruminococcaceae before tumor induction in the GE-fed diet group relative to the control group. After induction of tumor, there was a ten-fold increase in members of Desulfovibrionaceae family of bacteria, which are gram-negative anaerobes with the ability to restore sulfate [45].

A study suggested the existence of a butyrate-producing taxonomic core in healthy colon, primarily consisting of certain Lachnospiraceae and Ruminococcaceae family members [46]. Butyrate suppresses nuclear factor-B (Nf-κB) activation and interferon γ production and upregulates peroxisome proliferator-activated receptor γ (PPARγ), through the inhibition of histone deacetylases (HDAC) [47, 48]. Previously, our lab has shown that combined epigallocatechin gallate (EGCG) and sodium butyrate induced apoptosis in colon cancer cells by decreasing global CpG methylation and induction of histone H3 hyperacetylation [49].

**Fig 9:**
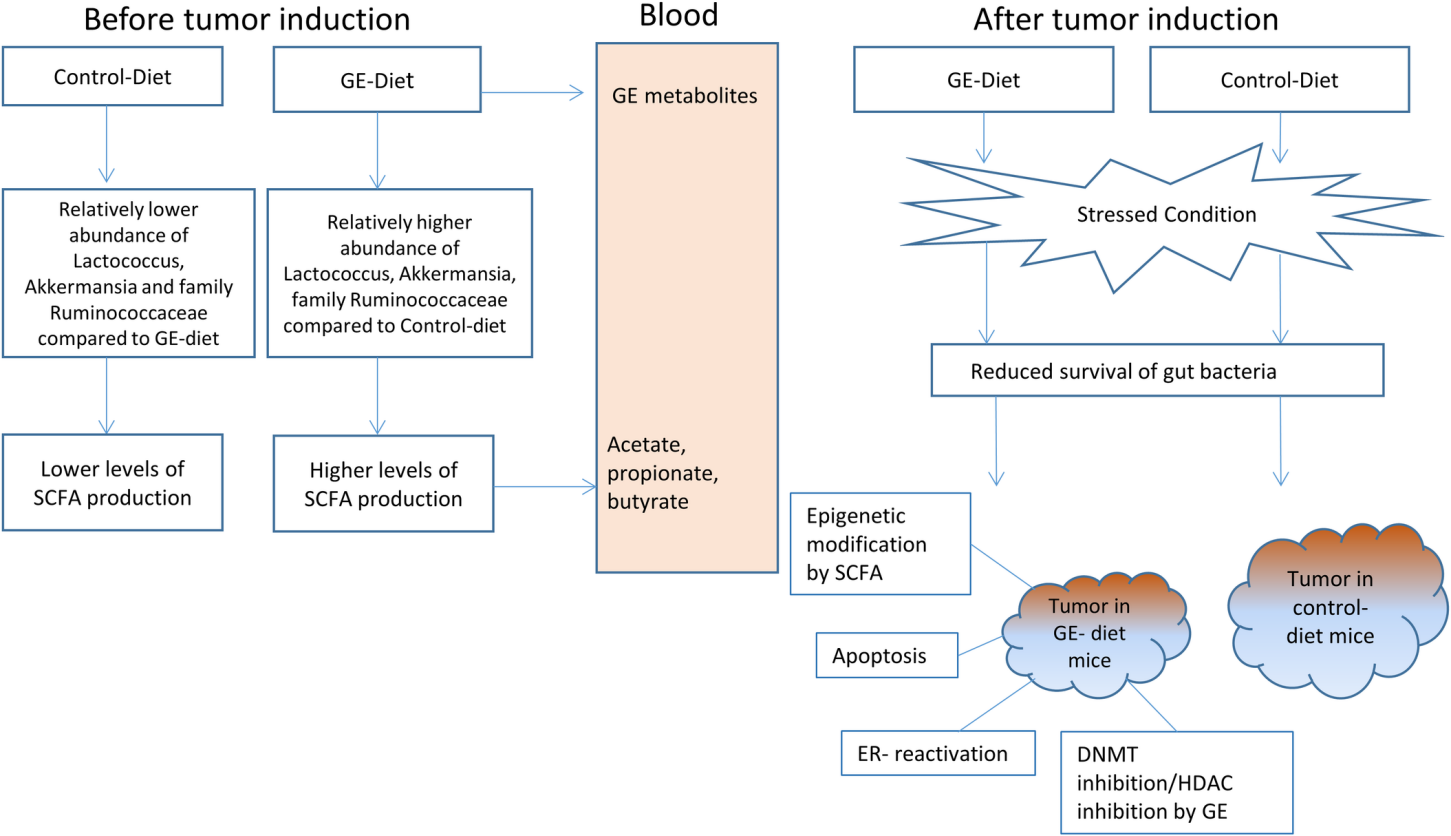
Hypothetical schematic for a mechanism of action of tumor reduction in GE-fed mice compared to control-fed mice. The figure shows a proposed mechanism for the observed changes in tumor reduction in GE-fed mice. The GE-fed mice had significantly different bacterial abundances as compared to control which may have caused an increase in SCFA synthesis. GE may have been metabolized by bacteria or otherwise into products that enhances the rate of apoptosis and delays the onset of tumor formation.

Future studies are warranted to determine the potential influence of intestinal microbiota on breast cancer risk that may be modulated via microbial effects on co-metabolism or metabolic exchange with the host. Certain microbiome members are not only directly pro-oncogenic but are capable of remodeling the intestinal lumen microbiota as a whole to promote progression of breast cancer. Previous studies have shown the effectiveness of GE in tumor reduction by acting as DNMT and HDAC inhibitors [11, 50]. The metabolites produced from bacteria that are abundant in GE-fed mice may play a positive role in increasing the latency of tumor by acting as epigenetic modulators. Our results represent a snapshot of the microbial structure of breast cancer patients before and after chemotherapy. A recent study has also found that patients with polycystic ovarian syndrome have lower gut diversity as compared to control [51]. Therefore, the role of gut microbiota in estrogen-driven diseases cannot be completely ruled out; however, a thorough investigation is required to study the impact of gut microbial changes on the metabolic profile. Larger studies are needed for detailed information concerning the pathways linking diet-microbiota shift and tumorigenesis. Fig 9 shows the hypothetical schematic of the proposed mechanism of reduction in tumor volume and weight in GE-fed mice as compared to control-fed mice.

Metabolomic studies have shown that gut bacteria cleave the heterocyclic ring of the isoflavonoid skeleton to produce 4-ethylphenol and 4-hydroxyphenyl-2-propionic acid [37], as well as reduce GE to dihydrogenistein [52]. In contrast in tissues, GE is metabolized mainly through oxidation, sulfation, glucuronidation, hydroxylation or methylation [53]. In the present study, LC-MS analysis of urines collected at the end of the study revealed that GE conjugates (sulfates and β-glucuronides) and heterocyclic ring cleaved products and their conjugates were equally prominent in mice humanized with fecal material from each of the three patients prior to chemotherapy. Since the urines were pooled from the four mice in each cage, it was not possible to assign statistical differences between the groups. Nonetheless, humanization with fecal material after chemotherapy caused a marked change in metabolism in two of three subjects with elimination of GE heterocyclic ring-cleaved products (4-ethylphenol and 2-(4-hydroxyphenyl)-propionic acid) and their conjugates. The effect of DOX on the microorganisms metabolizing GE was more extreme than docetaxel, potentially a reflection of its antibiotic properties as a member of the anthracycline family [54]. There was a large increase in GE sulfates and GE double conjugates in mice receiving fecal organisms from the patient after receiving DOX (G4 mouse group). This was not observed for mice humanized with the fecal material from the two patients treated with docetaxel and may reflect inhibition of catabolism of GE, resulting in increased amounts of phase II conjugates. With regard to when GE and its conjugates, or its ring-opened metabolites are the active agent in prevention of the growth of the injected MDA-MB-231 cells, a limitation of this study is that the urines were collected at the time of euthanasia and not during latency when the growth rate of the tumor cells was maximally inhibited. As noted earlier, although GE significantly changed the microbiome over a 4-week period, during the exposure to MDA-MB-231 mammary tumor cells, differences in the fecal microbiome largely dissipated. Understanding GE metabolism during the latency period may provide important insights to GE’s mechanism of action.

In summary, our results demonstrated successful humanization of germ-free mice by transplanting fecal samples from breast cancer patients. The microbiome of GE-fed mice had a distinct and different clustering compared to control-fed mice indicating an alteration of microbiota abundance and composition. The GE diet also resulted in increased latency in tumor induction, a significant reduction in tumor weight and a near-significant reduction in tumor volume. Our studies have shown that after consumption of GE diet, there was an increase in abundance of members of family Lachnospiraceae and Ruminococcaceae in mice. A recent study has shown that *in vitro* isoflavones promote SCFA production due to growth stimulation of SCFA-producing bacteria belonging to family Ruminococaceae and Lachnospiraceae [55]. We hypothesize that increased production of SCFAs in GE-fed mice may have induced epigenetic changes resulting in the reduction of tumor size and increased tumor latency. Future studies are warranted to explore how much of the reduction in tumor can be attributed to GE acting directly through blood and how much is due to the microbial shift and the metabolite production by some of the important bacteria of the gut.

## Supporting information

S1 Table Naming convention for heat map for pre-tumor data. Table shows the naming convention for heat map before induction of tumor as shown in Fig 5A. The table shows the kingdom, phylum, class, order, family, genus and species of the significantly different bacterial abundances between the GE-fed and control-fed mice groups.
(DOCX)

S2 Table Naming convention of heat map for post-tumor data. Table shows the naming convention for heat map after induction of tumor as shown in Fig 5B. The table shows the kingdom, phylum, class, order, family, genus and species of the significantly different bacterial abundances between the GE-fed and control-fed mice groups.
(DOCX)

S3 Table Comparison of pre-chemotherapy and post-chemotherapy bacterial abundances before induction of tumor. The table shows the comparison between bacterial abundances of pre-chemotherapy and post-chemotherapy group of mice, before induction of tumor and after introduction of genistein diet.
(DOCX)

S4 Table Comparison of pre-chemotherapy and post-chemotherapy bacterial abundances after induction of tumor. The table shows the comparison between bacterial abundances of pre-chemotherapy and post-chemotherapy group of mice, after induction of tumor.
(DOCX)

## References

[1] Siegel RL, Miller KD, Jemal A. Cancer statistics, 2016. CA Cancer J Clin. 2016;66(1):7–30. doi: 10.3322/caac.21332 26742998 10.3322/caac.21332

[2] Miller KD, Siegel RL, Lin CC, Mariotto AB, Kramer JL, Rowland JH, et al Cancer treatment and survivorship statistics, 2016. CA Cancer J Clin. 2016;66(4):271–89. doi: 10.3322/caac.21349 27253694 10.3322/caac.21349

[3] Crown J, O'Leary M, Ooi WS. Docetaxel and paclitaxel in the treatment of breast cancer: a review of clinical experience. Oncologist. 2004;9 Suppl 2:24–32. 15161988 10.1634/theoncologist.9-suppl_2-24

[4] Gho SA, Steele JR, Jones SC, Munro BJ. Self-reported side effects of breast cancer treatment: a cross-sectional study of incidence, associations, and the influence of exercise. Cancer Causes Control. 2013;24(3):517–28. doi: 10.1007/s10552-012-0142-4 23296457 10.1007/s10552-012-0142-4

[5] Saghatchian M, Bihan C, Chenailler C, Mazouni C, Dauchy S, Delaloge S. Exploring frontiers: use of complementary and alternative medicine among patients with early-stage breast cancer. Breast. 2014;23(3):279–85. doi: 10.1016/j.breast.2014.01.009 24529905 10.1016/j.breast.2014.01.009

[6] Chen H, Landen CN, Li Y, Alvarez RD, Tollefsbol TO. Enhancement of Cisplatin-Mediated Apoptosis in Ovarian Cancer Cells through Potentiating G2/M Arrest and p21 Upregulation by Combinatorial Epigallocatechin Gallate and Sulforaphane. Journal of oncology. 2013;2013:872957 doi: 10.1155/2013/872957 23476648 10.1155/2013/872957 PMC3588178

[7] Li Y, Meeran SM, Patel SN, Chen H, Hardy TM, Tollefsbol TO. Epigenetic reactivation of estrogen receptor-alpha (ERalpha) by genistein enhances hormonal therapy sensitivity in ERalpha-negative breast cancer. Mol Cancer. 2013;12:9 doi: 10.1186/1476-4598-12-9 23379261 10.1186/1476-4598-12-9 PMC3577460

[8] Andres S, Abraham K, Appel KE, Lampen A. Risks and benefits of dietary isoflavones for cancer. Critical reviews in toxicology. 2011;41(6):463–506. doi: 10.3109/10408444.2010.541900 21438720 10.3109/10408444.2010.541900

[9] Lee S-A, Shu X-O, Li H, Yang G, Cai H, Wen W, et al Adolescent and adult soy food intake and breast cancer risk: results from the Shanghai Women's Health Study. The American journal of clinical nutrition. 2009;89(6):1920–6. doi: 10.3945/ajcn.2008.27361 19403632 10.3945/ajcn.2008.27361 PMC2683002

[10] Mahmoud AM, Yang W, Bosland MC. Soy isoflavones and prostate cancer: a review of molecular mechanisms. J Steroid Biochem Mol Biol. 2014;140:116–32. doi: 10.1016/j.jsbmb.2013.12.010 24373791 10.1016/j.jsbmb.2013.12.010 PMC3962012

[11] Xie Q, Bai Q, Zou LY, Zhang QY, Zhou Y, Chang H, et al Genistein inhibits DNA methylation and increases expression of tumor suppressor genes in human breast cancer cells. Genes Chromosomes Cancer. 2014;53(5):422–31. doi: 10.1002/gcc.22154 24532317 10.1002/gcc.22154

[12] Li Y, Liu L, Andrews LG, Tollefsbol TO. Genistein depletes telomerase activity through cross-talk between genetic and epigenetic mechanisms. Int J Cancer. 2009;125(2):286–96. doi: 10.1002/ijc.24398 19358274 10.1002/ijc.24398 PMC2995334

[13] Muthyala RS, Ju YH, Sheng S, Williams LD, Doerge DR, Katzenellenbogen BS, et al Equol, a natural estrogenic metabolite from soy isoflavones: convenient preparation and resolution of R- and S-equols and their differing binding and biological activity through estrogen receptors alpha and beta. Bioorg Med Chem. 2004;12(6):1559–67. doi: 10.1016/j.bmc.2003.11.035 15018930 10.1016/j.bmc.2003.11.035

[14] Landete JM, Arques J, Medina M, Gaya P, de Las Rivas B, Munoz R. Bioactivation of Phytoestrogens: Intestinal Bacteria and Health. Crit Rev Food Sci Nutr. 2016;56(11):1826–43. doi: 10.1080/10408398.2013.789823 25848676 10.1080/10408398.2013.789823

[15] Mondot S, Lepage P. The human gut microbiome and its dysfunctions through the meta-omics prism. Ann N Y Acad Sci. 2016;1372(1):9–19. doi: 10.1111/nyas.13033 26945826 10.1111/nyas.13033

[16] Sender R, Fuchs S, Milo R. Revised estimates for the number of human and bacteria cells in the body. PLoS biology. 2016;14(8):e1002533 doi: 10.1371/journal.pbio.1002533 27541692 10.1371/journal.pbio.1002533 PMC4991899

[17] Paul B, Barnes S, Demark-Wahnefried W, Morrow C, Salvador C, Skibola C, et al Influences of diet and the gut microbiome on epigenetic modulation in cancer and other diseases. Clin Epigenetics. 2015;7:112 doi: 10.1186/s13148-015-0144-7 26478753 10.1186/s13148-015-0144-7 PMC4609101

[18] Louis P, Hold GL, Flint HJ. The gut microbiota, bacterial metabolites and colorectal cancer. Nat Rev Microbiol. 2014;12(10):661–72. doi: 10.1038/nrmicro3344 25198138 10.1038/nrmicro3344

[19] Goedert JJ, Jones G, Hua X, Xu X, Yu G, Flores R, et al Investigation of the Association Between the Fecal Microbiota and Breast Cancer in Postmenopausal Women: a Population-Based Case-Control Pilot Study. JNCI: Journal of the National Cancer Institute. 2015;107(8):djv147–djv. doi: 10.1093/jnci/djv147 26032724 10.1093/jnci/djv147 PMC4554191

[20] Liang Q, Chiu J, Chen Y, Huang Y, Higashimori A, Fang J, et al Fecal Bacteria Act as Novel Biomarkers for Noninvasive Diagnosis of Colorectal Cancer. Clin Cancer Res. 2017;23(8):2061–70. doi: 10.1158/1078-0432.CCR-16-1599 27697996 10.1158/1078-0432.CCR-16-1599

[21] Panasevich MR, Schuster CM, Phillips KE, Meers GM, Chintapalli SV, Wankhade UD, et al Soy compared with milk protein in a Western diet changes fecal microbiota and decreases hepatic steatosis in obese OLETF rats. The Journal of Nutritional Biochemistry. 2017. 10.1016/j.jnutbio.2017.05.004 PMC5542587 28605664

[22] Huang H, Roman Arocho GM, Davis C, Yu L, Wang TTY. Consumption of Selected Cruciferous Vegetables and Soy Phytochemical Dietary Supplements Can Alter Gut Microbiome Composition. The FASEB Journal. 2016;30(1 Supplement):1176 26.

[23] Wilson ID, Nicholson JK. Gut microbiome interactions with drug metabolism, efficacy, and toxicity. Transl Res. 2017;179:204–22. doi: 10.1016/j.trsl.2016.08.002 27591027 10.1016/j.trsl.2016.08.002 PMC5718288

[24] Cary SG, Blair EB. New Transport Medium for Shipment of Clinical Specimens. I. Fecal Specimens. J Bacteriol. 1964;88:96–8. 14197911 10.1128/jb.88.1.96-98.1964 PMC277262

[25] Kumar R, Eipers P, Little RB, Crowley M, Crossman DK, Lefkowitz EJ, et al Getting started with microbiome analysis: sample acquisition to bioinformatics. Current Protocols in Human Genetics. 2014:18 8. 1–8. 29. 10.1002/0471142905.hg1808s82 PMC4383038 25042718

[26] Caporaso JG, Lauber CL, Walters WA, Berg-Lyons D, Huntley J, Fierer N, et al Ultra-high-throughput microbial community analysis on the Illumina HiSeq and MiSeq platforms. The ISME journal. 2012;6(8):1621 doi: 10.1038/ismej.2012.8 22402401 10.1038/ismej.2012.8 PMC3400413

[27] Caporaso JG, Lauber CL, Walters WA, Berg-Lyons D, Lozupone CA, Turnbaugh PJ, et al Global patterns of 16S rRNA diversity at a depth of millions of sequences per sample. Proc Natl Acad Sci U S A. 2011;108 Suppl 1:4516–22. 20534432 10.1073/pnas.1000080107 PMC3063599

[28] DeSantis TZ, Hugenholtz P, Larsen N, Rojas M, Brodie EL, Keller K, et al Greengenes, a chimera-checked 16S rRNA gene database and workbench compatible with ARB. Appl Environ Microbiol. 2006;72(7):5069–72. doi: 10.1128/AEM.03006-05 16820507 10.1128/AEM.03006-05 PMC1489311

[29] Caporaso JG, Bittinger K, Bushman FD, DeSantis TZ, Andersen GL, Knight R. PyNAST: a flexible tool for aligning sequences to a template alignment. Bioinformatics. 2010;26(2):266–7. doi: 10.1093/bioinformatics/btp636 19914921 10.1093/bioinformatics/btp636 PMC2804299

[30] Chao A, Colwell RK, Lin C-W, Gotelli NJ. Sufficient sampling for asymptotic minimum species richness estimators. Ecology. 2009;90(4):1125–33. 19449706 10.1890/07-2147.1

[31] Keylock C. Simpson diversity and the Shannon–Wiener index as special cases of a generalized entropy. Oikos. 2005;109(1):203–7.

[32] Faith DP, Baker AM. Phylogenetic diversity (PD) and biodiversity conservation: some bioinformatics challenges. Evol Bioinform Online. 2007;2:121–8. 19455206 PMC2674678

[33] Lozupone C, Knight R. UniFrac: a new phylogenetic method for comparing microbial communities. Appl Environ Microbiol. 2005;71(12):8228–35. doi: 10.1128/AEM.71.12.8228-8235.2005 16332807 10.1128/AEM.71.12.8228-8235.2005 PMC1317376

[34] Beals EW. Bray-Curtis ordination: an effective strategy for analysis of multivariate ecological data. Advances in ecological research. 1984;14:1–55.

[35] Kumar R, Maynard CL, Eipers P, Goldsmith KT, Ptacek T, Grubbs JA, et al Colonization potential to reconstitute a microbe community in patients detected early after fecal microbe transplant for recurrent C. difficile. BMC microbiology. 2016;16(1):5. 26758906 10.1186/s12866-015-0622-2 PMC4711103

[36] Camacho L, Lewis SM, Vanlandingham MM, Juliar BE, Olson GR, Patton RE, et al Comparison of endpoints relevant to toxicity assessments in 3 generations of CD-1 mice fed irradiated natural and purified ingredient diets with varying soy protein and isoflavone contents. Food and Chemical Toxicology. 2016;94:39–56. doi: 10.1016/j.fct.2016.05.014 27234134 10.1016/j.fct.2016.05.014 PMC4930898

[37] Coldham NG, Howells LC, Santi A, Montesissa C, Langlais C, King LJ, et al Biotransformation of genistein in the rat: elucidation of metabolite structure by product ion mass fragmentology n. The Journal of steroid biochemistry and molecular biology. 1999;70(4):169–84. 10622405 10.1016/s0960-0760(99)00104-1

[38] Soukup ST, Helppi J, Müller DR, Zierau O, Watzl B, Vollmer G, et al Phase II metabolism of the soy isoflavones genistein and daidzein in humans, rats and mice: a cross-species and sex comparison. Archives of toxicology. 2016;90(6):1335–47. doi: 10.1007/s00204-016-1663-5 26838042 10.1007/s00204-016-1663-5

[39] Clavel T, Lagkouvardos I, Blaut M, Stecher B. The mouse gut microbiome revisited: From complex diversity to model ecosystems. Int J Med Microbiol. 2016;306(5):316–27. doi: 10.1016/j.ijmm.2016.03.002 26995267 10.1016/j.ijmm.2016.03.002

[40] Turnbaugh PJ, Ridaura VK, Faith JJ, Rey FE, Knight R, Gordon JI. The effect of diet on the human gut microbiome: a metagenomic analysis in humanized gnotobiotic mice. Sci Transl Med. 2009;1(6):6ra14 doi: 10.1126/scitranslmed.3000322 20368178 10.1126/scitranslmed.3000322 PMC2894525

[41] Mazmanian SK, Round JL, Kasper DL. A microbial symbiosis factor prevents intestinal inflammatory disease. Nature. 2008;453(7195):620–5. doi: 10.1038/nature07008 18509436 10.1038/nature07008

[42] Baker JM, Al-Nakkash L, Herbst-Kralovetz MM. Estrogen–gut microbiome axis: Physiological and clinical implications. Maturitas. 2017;103(Supplement C):45–53. 28778332 10.1016/j.maturitas.2017.06.025

[43] Kim SY, Kim JE, Lee KW, Lee HJ. Lactococcus lactis ssp. lactis inhibits the proliferation of SNU-1 human stomach cancer cells through induction of G0/G1 cell cycle arrest and apoptosis via p53 and p21 expression. Ann N Y Acad Sci. 2009;1171:270–5. doi: 10.1111/j.1749-6632.2009.04721.x 19723065 10.1111/j.1749-6632.2009.04721.x

[44] Fernandes A, Costa R, Gaspar P, Neves A, Vinga S, editors. Dynamic Flux Balance Analysis for Modeling Lactococcus lactis Mannitol Production. 9th European Conference on Mathematical and Theoretical Biology; 2014.

[45] Kertesz MA. Riding the sulfur cycle–metabolism of sulfonates and sulfate esters in Gram-negative bacteria. FEMS Microbiology Reviews. 2000;24(2):135–75. 10717312 10.1016/S0168-6445(99)00033-9

[46] Vital M, Howe AC, Tiedje JM. Revealing the bacterial butyrate synthesis pathways by analyzing (meta) genomic data. MBio. 2014;5(2):e00889–14. doi: 10.1128/mBio.00889-14 24757212 10.1128/mBio.00889-14 PMC3994512

[47] Hamer HM, Jonkers D, Venema K, Vanhoutvin S, Troost F, Brummer RJ. The role of butyrate on colonic function. Alimentary pharmacology & therapeutics. 2008;27(2):104–19. 17973645 10.1111/j.1365-2036.2007.03562.x

[48] Kim MH, Kang SG, Park JH, Yanagisawa M, Kim CH. Short-chain fatty acids activate GPR41 and GPR43 on intestinal epithelial cells to promote inflammatory responses in mice. Gastroenterology. 2013;145(2):396–406. e10. doi: 10.1053/j.gastro.2013.04.056 23665276 10.1053/j.gastro.2013.04.056

[49] Saldanha SN, Kala R, Tollefsbol TO. Molecular mechanisms for inhibition of colon cancer cells by combined epigenetic-modulating epigallocatechin gallate and sodium butyrate. Experimental cell research. 2014;324(1):40–53. doi: 10.1016/j.yexcr.2014.01.024 24518414 10.1016/j.yexcr.2014.01.024 PMC4043227

[50] de Cremoux P, Dalvai M, N’Doye O, Moutahir F, Rolland G, Chouchane-Mlik O, et al HDAC inhibition does not induce estrogen receptor in human triple-negative breast cancer cell lines and patient-derived xenografts. Breast cancer research and treatment. 2015;149(1):81–9. doi: 10.1007/s10549-014-3233-y 25503779 10.1007/s10549-014-3233-y

[51] Lindheim L, Bashir M, Münzker J, Trummer C, Zachhuber V, Leber B, et al Alterations in Gut Microbiome Composition and Barrier Function Are Associated with Reproductive and Metabolic Defects in Women with Polycystic Ovary Syndrome (PCOS): A Pilot Study. PLOS ONE. 2017;12(1):e0168390 doi: 10.1371/journal.pone.0168390 28045919 10.1371/journal.pone.0168390 PMC5207627

[52] Spagnuolo C, Russo GL, Orhan IE, Habtemariam S, Daglia M, Sureda A, et al Genistein and cancer: current status, challenges, and future directions. Advances in Nutrition: An International Review Journal. 2015;6(4):408–19. 10.3945/an.114.008052 PMC4496735 26178025

[53] Nguyen DT, Hernandez-Montes E, Vauzour D, Schönthal AH, Rice-Evans C, Cadenas E, et al The intracellular genistein metabolite 5, 7, 3′, 4′-tetrahydroxyisoflavone mediates G2-M cell cycle arrest in cancer cells via modulation of the p38 signaling pathway. Free Radical Biology and Medicine. 2006;41(8):1225–39. doi: 10.1016/j.freeradbiomed.2006.06.026 17015169 10.1016/j.freeradbiomed.2006.06.026

[54] Arcamone F, Cassinelli G, Fantini G, Grein A, Orezzi P, Pol C, et al Adriamycin, 14‐Hydroxydaunomycin, a new antitumor antibiotic from S. peucetius var. caesius. Biotechnology and bioengineering. 2000;67(6):704–13. 10699851 10.1002/(sici)1097-0290(20000320)67:6<704::aid-bit8>3.0.co;2-l

[55] Guadamuro L, Dohrmann AB, Tebbe CC, Mayo B, Delgado S. Bacterial communities and metabolic activity of faecal cultures from equol producer and non-producer menopausal women under treatment with soy isoflavones. BMC microbiology. 2017;17(1):93 doi: 10.1186/s12866-017-1001-y 28415978 10.1186/s12866-017-1001-y PMC5392999

