# Supplementary material for: Impact of genistein on the gut microbiome of humanized mice and its role in breast tumor inhibition
Source: PLoS One. 2017 Dec 21;12(12):e0189756. doi: 10.1371/journal.pone.0189756 (PMC5739415; doi:10.1371/journal.pone.0189756)
Supplement: S2 Table — Table shows the naming convention for heat map after induction of tumor as shown in Fig 5B. The table shows the kingdom, phylum, class, order, family, genus and species of the significantly different bacterial abundances between the GE-fed and control-fed mice groups. (DOCX) [file pone.0189756.s002.docx]

**S2 Table: Naming convention for post-tumor data**

| **Naming (Heatmap)** | **Kingdom** | **Phylum** | **Class** | **Order** | **Family** | **Genus** | **Species** |
| --- | --- | --- | --- | --- | --- | --- | --- |
| Verr_Akke_muci | Bacteria | Verrucomicrobia | Verrucomicrobiae | Verrucomicrobiales | Verrucomicrobiaceae | Akkermansia | muciniphila |
| Bact_Bact_ | Bacteria | Bacteroidetes | Bacteroidia | Bacteroidales | Bacteroidaceae | Bacteroides |  |
| Bact_Bact_unif | Bacteria | Bacteroidetes | Bacteroidia | Bacteroidales | Bacteroidaceae | Bacteroides | uniformis |
| Bact_Bact_frag | Bacteria | Bacteroidetes | Bacteroidia | Bacteroidales | Bacteroidaceae | Bacteroides | fragilis |
| Lact_Lact_ | Bacteria | Firmicutes | Bacilli | Lactobacillales | Lactobacillaceae | Lactobacillus |  |
| Bact_Bact_ | Bacteria | Bacteroidetes | Bacteroidia | Bacteroidales | Bacteroidaceae | Bacteroides |  |
| Ente_Ente_ | Bacteria | Firmicutes | Bacilli | Lactobacillales | Enterococcaceae | Enterococcus |  |
| Bact_Bact_cacc | Bacteria | Bacteroidetes | Bacteroidia | Bacteroidales | Bacteroidaceae | Bacteroides | caccae |
| Rumi__ | Bacteria | Firmicutes | Clostridia | Clostridiales | Ruminococcaceae |  |  |
| Lach_Blau_ | Bacteria | Firmicutes | Clostridia | Clostridiales | Lachnospiraceae | Blautia |  |
| Lach__ | Bacteria | Firmicutes | Clostridia | Clostridiales | Lachnospiraceae |  |  |
| Alca_Sutt_ | Bacteria | Proteobacteria | Betaproteobacteria | Burkholderiales | Alcaligenaceae | Sutterella |  |
| Erys__ | Bacteria | Firmicutes | Erysipelotrichi | Erysipelotrichales | Erysipelotrichaceae |  |  |
| [Bar_ _ | Bacteria | Bacteroidetes | Bacteroidia | Bacteroidales | [Barnesiellaceae] |  |  |
| [Odo_Buty_ | Bacteria | Bacteroidetes | Bacteroidia | Bacteroidales | [Odoribacteraceae] | Butyricimonas |  |
| Lach_[Rum_torq | Bacteria | Firmicutes | Clostridia | Clostridiales | Lachnospiraceae | [Ruminococcus] | torques |
| Lach_Blau_prod | Bacteria | Firmicutes | Clostridia | Clostridiales | Lachnospiraceae | Blautia | producta |
| Lach_Dore_ | Bacteria | Firmicutes | Clostridia | Clostridiales | Lachnospiraceae | Dorea |  |
| Rike_ _ | Bacteria | Bacteroidetes | Bacteroidia | Bacteroidales | Rikenellaceae |  |  |
| Desu_Bilo_ | Bacteria | Proteobacteria | Deltaproteobacteria | Desulfovibrionales | Desulfovibrionaceae | Bilophila |  |
| Bact_Bact_ | Bacteria | Bacteroidetes | Bacteroidia | Bacteroidales | Bacteroidaceae | Bacteroides |  |
| Pept_Pept_ | Bacteria | Firmicutes | Clostridia | Clostridiales | Peptococcaceae | Peptococcus |  |
| Lach_Dore_ | Bacteria | Firmicutes | Clostridia | Clostridiales | Lachnospiraceae | Dorea |  |
| Lach_ _ | Bacteria | Firmicutes | Clostridia | Clostridiales | Lachnospiraceae |  |  |
| Veil_Phas_ | Bacteria | Firmicutes | Clostridia | Clostridiales | Veillonellaceae | Phascolarctobacterium |  |
