# Supplementary material for: Impact of genistein on the gut microbiome of humanized mice and its role in breast tumor inhibition
Source: PLoS One. 2017 Dec 21;12(12):e0189756. doi: 10.1371/journal.pone.0189756 (PMC5739415; doi:10.1371/journal.pone.0189756)
Supplement: S3 Table — The table shows the comparison between bacterial abundances of pre-chemotherapy and post-chemotherapy group of mice, before induction of tumor and after introduction of genistein diet. (DOCX) [file pone.0189756.s003.docx]

**S3 Table: Bacterial species showing differences between pre-chemotherapy and post-chemotherapy before tumor induction**

| **Phylum** | **Class** | **Order** | **Family** | **Genus** | **Species** | **t-test *p*-value** |
| --- | --- | --- | --- | --- | --- | --- |
| Verrucomicrobia | Verrucomicrobiae | Verrucomicrobiales | Verrucomicrobiaceae | Akkermansia | muciniphila | 0.231524898 |
| Bacteroidetes | Bacteroidia | Bacteroidales | Bacteroidaceae | Bacteroides |  | 0.296162894 |
| Bacteroidetes | Bacteroidia | Bacteroidales | Bacteroidaceae | Bacteroides | uniformis | 0.438868304 |
| Bacteroidetes | Bacteroidia | Bacteroidales | Bacteroidaceae | Bacteroides | eggerthii | 0.726922466 |
| Bacteroidetes | Bacteroidia | Bacteroidales | Bacteroidaceae | Bacteroides |  | 0.951736232 |
| Firmicutes | Clostridia | Clostridiales | Lachnospiraceae | Blautia |  | 0.758744088 |
| Bacteroidetes | Bacteroidia | Bacteroidales | Bacteroidaceae | Bacteroides | caccae | 0.086998351 |
| Bacteroidetes | Bacteroidia | Bacteroidales | Bacteroidaceae | Bacteroides |  | 0.469726453 |
| Firmicutes | Clostridia | Clostridiales | Lachnospiraceae |  |  | 0.174378041 |
| Firmicutes | Erysipelotrichi | Erysipelotrichales | Erysipelotrichaceae |  |  | 0.270739996 |
| Firmicutes | Bacilli | Lactobacillales | Lactobacillaceae | Lactobacillus |  | 0.823327352 |
| Firmicutes | Bacilli | Turicibacterales | Turicibacteraceae | Turicibacter |  | 0.963887207 |
| Firmicutes | Clostridia | Clostridiales | Lachnospiraceae | Blautia | producta | 0.93187326 |
| Firmicutes | Clostridia | Clostridiales | Ruminococcaceae |  |  | 0.186955031 |
| Proteobacteria | Betaproteobacteria | Burkholderiales | Alcaligenaceae | Sutterella |  | 0.396391393 |
| Firmicutes | Clostridia | Clostridiales | Lachnospiraceae |  |  | 0.554496188 |
| Bacteroidetes | Bacteroidia | Bacteroidales | [Odoribacteraceae] | Butyricimonas |  | 0.202416359 |
| Bacteroidetes | Bacteroidia | Bacteroidales | Bacteroidaceae | Bacteroides | ovatus | 0.276627527 |
| Firmicutes | Clostridia | Clostridiales | Lachnospiraceae |  |  | 0.523812316 |
| Bacteroidetes | Bacteroidia | Bacteroidales | Porphyromonadaceae | Parabacteroides |  | 0.420669295 |
| Firmicutes | Clostridia | Clostridiales | Veillonellaceae | Phascolarctobacterium |  | 0.552324535 |
| Firmicutes | Clostridia | Clostridiales | Clostridiaceae |  |  | 0.250962233 |
| Verrucomicrobia | Verrucomicrobiae | Verrucomicrobiales | Verrucomicrobiaceae | Akkermansia | muciniphila | 0.206390964 |
| Firmicutes | Clostridia | Clostridiales | Lachnospiraceae | Clostridium | citroniae | 0.728289166 |
| Firmicutes | Clostridia | Clostridiales | Lachnospiraceae | Dorea |  | 0.183006098 |
