# Supplementary material for: Impact of genistein on the gut microbiome of humanized mice and its role in breast tumor inhibition
Source: PLoS One. 2017 Dec 21;12(12):e0189756. doi: 10.1371/journal.pone.0189756 (PMC5739415; doi:10.1371/journal.pone.0189756)
Supplement: S4 Table — The table shows the comparison between bacterial abundances of pre-chemotherapy and post-chemotherapy group of mice, after induction of tumor. (DOCX) [file pone.0189756.s004.docx]

**S4 Table: Bacterial species showing differences between pre-chemotherapy and post-chemotherapy after tumor induction**

| **Phylum** | **Class** | **Order** | **Family** | **Genus** | **Species** | **t-test *p*-value** |
| --- | --- | --- | --- | --- | --- | --- |
| Verrucomicrobia | Verrucomicrobiae | Verrucomicrobiales | Verrucomicrobiaceae | Akkermansia | muciniphila | 0.233307789 |
| Bacteroidetes | Bacteroidia | Bacteroidales | Bacteroidaceae | Bacteroides |  | 0.959041684 |
| Bacteroidetes | Bacteroidia | Bacteroidales | Bacteroidaceae | Bacteroides | uniformis | 0.917437396 |
| Bacteroidetes | Bacteroidia | Bacteroidales | Bacteroidaceae | Bacteroides | fragilis | 0.270154949 |
| Firmicutes | Bacilli | Lactobacillales | Lactobacillaceae | Lactobacillus |  | 0.077470831 |
| Bacteroidetes | Bacteroidia | Bacteroidales | Bacteroidaceae | Bacteroides |  | 0.419777982 |
| Firmicutes | Bacilli | Lactobacillales | Enterococcaceae | Enterococcus |  | 0.620187051 |
| Bacteroidetes | Bacteroidia | Bacteroidales | Bacteroidaceae | Bacteroides | caccae | 0.563583721 |
| Firmicutes | Clostridia | Clostridiales | Ruminococcaceae |  |  | 0.550743081 |
| Firmicutes | Clostridia | Clostridiales | Lachnospiraceae | Blautia |  | 0.722690508 |
| Firmicutes | Clostridia | Clostridiales | Lachnospiraceae |  |  | 0.220519666 |
| Proteobacteria | Betaproteobacteria | Burkholderiales | Alcaligenaceae | Sutterella |  | 0.331784339 |
| Firmicutes | Erysipelotrichi | Erysipelotrichales | Erysipelotrichaceae |  |  | 0.333162202 |
| Bacteroidetes | Bacteroidia | Bacteroidales | [Barnesiellaceae] |  |  | 0.274248566 |
| Bacteroidetes | Bacteroidia | Bacteroidales | [Odoribacteraceae] | Butyricimonas |  | 0.510558621 |
| Firmicutes | Clostridia | Clostridiales | Lachnospiraceae | [Ruminococcus] | torques | 0.642279864 |
| Firmicutes | Clostridia | Clostridiales | Lachnospiraceae | Blautia | producta | 0.602651185 |
| Firmicutes | Clostridia | Clostridiales | Lachnospiraceae | Dorea |  | 0.297176628 |
| Bacteroidetes | Bacteroidia | Bacteroidales | Rikenellaceae |  |  | 0.488906146 |
| Proteobacteria | Deltaproteobacteria | Desulfovibrionales | Desulfovibrionaceae | Bilophila |  | 0.837690585 |
| Bacteroidetes | Bacteroidia | Bacteroidales | Bacteroidaceae | Bacteroides |  | 0.191144867 |
| Firmicutes | Clostridia | Clostridiales | Peptococcaceae | Peptococcus |  | 0.374223173 |
| Firmicutes | Clostridia | Clostridiales | Lachnospiraceae | Dorea |  | 0.346859784 |
| Firmicutes | Clostridia | Clostridiales | Lachnospiraceae |  |  | 0.273768269 |
| Firmicutes | Clostridia | Clostridiales | Veillonellaceae | Phascolarctobacterium |  | 0.973266399 |
